# A randomized controlled trial of dihydroartemisinin-piperaquine, artesunate-mefloquine and extended artemether-lumefantrine treatments for malaria in pregnancy on the Thailand-Myanmar border

**DOI:** 10.1186/s12916-021-02002-8

**Published:** 2021-06-10

**Authors:** Makoto Saito, Verena I. Carrara, Mary Ellen Gilder, Aung Myat Min, Nay Win Tun, Mupawjay Pimanpanarak, Jacher Viladpai-nguen, Moo Kho Paw, Warat Haohankhunnatham, Kamonchanok Konghahong, Aung Pyae Phyo, Cindy Chu, Claudia Turner, Sue J. Lee, Jureeporn Duanguppama, Mallika Imwong, Germana Bancone, Stephane Proux, Pratap Singhasivanon, Nicholas J. White, François Nosten, Rose McGready

**Affiliations:** 1 Shoklo Malaria Research Unit, Mahidol-Oxford Tropical Medicine Research Unit, Faculty of Tropical Medicine, Mahidol University, Mae Sot, Thailand; 2 Centre for Tropical Medicine and Global Health, Nuffield Department of Medicine, University of Oxford, Oxford, UK; 3 Division of Infectious Diseases, Advanced Clinical Research Center, Institute of Medical Science, University of Tokyo, Tokyo, Japan; 4 Institute of Global Health, Faculty of Medicine, University of Geneva, Geneva, Switzerland; 5 Department of Family Medicine, Chiang Mai University, Chiang Mai, Thailand; 6 Mahidol-Oxford Tropical Medicine Research Unit (MORU), Faculty of Tropical Medicine, Mahidol University, Bangkok, Thailand; 7 Department of Molecular Tropical Medicine and Genetics, Faculty of Tropical Medicine, Mahidol University, Bangkok, Thailand; 8 Department of Tropical Hygiene, Faculty of Tropical Medicine, Mahidol University, Bangkok, Thailand

**Keywords:** Malaria, *Plasmodium falciparum*, *Plasmodium vivax*, Pregnancy, Artemisinin-based combination therapy, Efficacy, Tolerability, Safety, *Pfkelch13*, *pfmdr1*

## Abstract

**Background:**

Artemisinin and artemisinin-based combination therapy (ACT) partner drug resistance in *Plasmodium falciparum* have spread across the Greater Mekong Subregion compromising antimalarial treatment. The current 3-day artemether-lumefantrine regimen has been associated with high treatment failure rates in pregnant women. Although ACTs are recommended for treating *Plasmodium vivax* malaria, no clinical trials in pregnancy have been reported.

**Methods:**

Pregnant women with uncomplicated malaria on the Thailand-Myanmar border participated in an open-label randomized controlled trial comparing dihydroartemisinin-piperaquine (DP), artesunate-mefloquine (ASMQ) and a 4-day artemether-lumefantrine regimen (AL^+^). The primary endpoint for *P. falciparum* infections was the PCR-corrected cure rate and for *P. vivax* infections was recurrent parasitaemia, before delivery or day 63, whichever was longer, assessed by Kaplan-Meier estimate.

**Results:**

Between February 2010 and August 2016, 511 pregnant women with malaria (353 *P. vivax*, 142 *P. falciparum*, 15 co-infections, 1 *Plasmodium malariae*) were randomized to either DP (*n*=170), ASMQ (*n*=169) or AL^+^ (*n*=172) treatments. Successful malaria elimination efforts in the region resulted in premature termination of the trial. The majority of women had recurrent malaria (mainly *P. vivax* relapses, which are not prevented by these treatments). Recurrence-free proportions (95% confidence interval [95% CI]) for vivax malaria were 20.6% (5.1–43.4) for DP (*n*=125), 46.0% (30.9–60.0) for ASMQ (*n*=117) and 28.7% (10.0–50.8) for AL^+^ (*n*=126). DP and ASMQ provided longer recurrence-free intervals. PCR-corrected cure rates (95% CI) for falciparum malaria were 93.7% (81.6–97.9) for DP (*n*=49), 79.6% (66.1–88.1) for AMSQ (*n*=55) and 87.5% (74.3–94.2) for AL^+^ (*n*=50). Overall 65% (85/130) of *P. falciparum* infections had *Pfkelch13* propeller mutations which increased over time and recrudescence occurred almost exclusively in them; risk ratio 9.42 (95% CI 1.30–68.29). Among the women with falciparum malaria, 24.0% (95% CI 16.8–33.6) had *P. vivax* parasitaemia within 4 months. Nausea, vomiting, dizziness and sleep disturbance were more frequent with ASMQ. Miscarriage, small-for-gestational-age and preterm birth did not differ significantly among the treatment groups, including first trimester exposures (*n*=46).

**Conclusions:**

DP was well tolerated and safe, and was the only drug providing satisfactory efficacy for *P. falciparum*-infected pregnant woman in this area of widespread artemisinin resistance. Vivax malaria recurrences are very common and warrant chloroquine prophylaxis after antimalarial treatment in this area.

**Trial registration:**

ClinicalTrials.gov identifier NCT01054248, registered on 22 January 2010.

**Supplementary Information:**

The online version contains supplementary material available at 10.1186/s12916-021-02002-8.

## Background

Malaria contributes substantially to poor pregnancy outcomes in tropical areas including miscarriage, preterm birth, stillbirth, small for gestational age (SGA) at birth, and at worst, maternal mortality [1, 2]. Chemotherapeutic approaches to malaria control in pregnancy include intermittent preventive treatment with sulfadoxine-pyrimethamine in sub-Saharan African countries with moderate to high malaria transmission, and regular screening and treatment at antenatal visits in other areas where transmission is generally lower, sulfadoxine-pyrimethamine resistance is higher, and *Plasmodium vivax* also is prevalent.

Artemisinin-based combination therapy (ACT) is recommended as the first-line treatment for both falciparum and non-falciparum malaria [3]. In pregnancy, ACTs have superior efficacy, effectiveness and tolerability compared with quinine in the treatment of falciparum malaria [4] and are considered safe during pregnancy, including in the first trimester; previous meta-analyses of clinical studies showed that artemisinin in the first trimester was not associated with increased risks of miscarriage, stillbirth or congenital abnormality compared with quinine [5–9].

Malaria treatment in South-East Asia is complicated by drug resistance, which severely limits treatment and excludes chemoprophylaxis in pregnancy for *Plasmodium falciparum*. Multiple drug resistance in *P. falciparum* is widespread, and in Indonesia and Oceania, *P. vivax* is often highly resistant to chloroquine [10]. The low efficacy of artemether-lumefantrine treatment of uncomplicated falciparum in pregnancy on the Thailand-Myanmar border (81.2%) reported previously was associated with low plasma lumefantrine concentrations [11, 12], suggesting that an extended dosing regimen might have improved efficacy. In the past 15 years, artemisinin and partner drug resistance has spread across the Greater Mekong Subregion (GMS). While alternatives (e.g. the triple ACTs) were tested [13], pregnant women were specifically excluded from these trials. For vivax malaria, radical cure to prevent relapses is contraindicated in pregnancy, so recurrences of vivax malaria are common and, although they may be delayed, these relapses are not prevented by the blood stage treatment. Indeed, there are no randomized controlled trials (RCTs) comparing the efficacy of different ACTs in pregnancy for the treatment of falciparum malaria in South-East Asia or *P. vivax* malaria anywhere, and none in women in the first trimester of pregnancy [4].

The primary aim of this study was to compare the efficacy, tolerability and pregnancy outcomes of three different ACT regimens in the treatment of uncomplicated malaria (of any species), on the Thailand-Myanmar border, part of GMS.

## Methods

### Participants

#### Study settings

The study participants attended one of three antenatal care clinics (ANCs) on the border between Thailand and Myanmar. Blood smears to detect malaria at ANC visits were offered twice per month, and treatment for the women with positive tests was provided regardless of symptoms. Haematocrit was measured and women received anaemia prophylaxis with ferrous sulphate (200 mg daily) and folic acid (5 mg once weekly) at each weekly visit. If women were anaemic, they received treatment with ferrous sulphate (200 mg thrice per day) and folic acid (5 mg daily) for 12 weeks. Gestational age was estimated by ultrasound at first ANC visit, or retrospectively by the Dubowitz examination at birth for those with an initial ultrasound later than 24 weeks of gestation.

#### Eligibility

The initial protocol included women aged 18–45 years, who were not in labour and were willing to comply with the protocol, and who had a viable second or third trimester pregnancy (confirmed by ultrasound examination) and acute uncomplicated malaria, including asymptomatic parasitaemia. When evidence supporting safety of artemisinins in the first trimester was published [14], a protocol amendment was granted in 2012 to include first trimester pregnancies with an ultrasound-detected foetal heartbeat. Women with an allergy to any of the drugs, severe malaria [3], severe anaemia and/or hyperparasitaemia (≥ 40 parasites per 1000 red blood cells) [3], or significant comorbidities were excluded. Written informed consent was obtained before entry to the study.

### Interventions

#### Antimalarial drug treatments and clinical management

Women were randomized to receive one of three ACTs: standard dose dihydroartemisinin-piperaquine (DP) and artesunate-mefloquine (ASMQ), and an extended artemether-lumefantrine (AL^+^) regimen. DP (Holley Pharmacy, China) was given as 2.4 mg/kg dihydroartemisinin with 20 mg/kg piperaquine once daily for 3 days, rounded to the nearest half tablet (40 mg/320 mg dihydroartemisinin/piperaquine per tablet). ASMQ was given once daily for 3 days, either as separate doses of artesunate 4 mg/kg/day and mefloquine 8.3 mg/kg/day, or fixed dose (artesunate 200 mg with mefloquine hydrochloride 440 mg each day: Far-Manguinhos, Brazil). The loose dose was rounded to the nearest quarter of a tablet for artesunate (50 mg/tablet: Guilin, China) and mefloquine (250 mg/tablet: Atlantic Laboratories Corp, Thailand). AL^+^ was given as five tablets (20/120 mg artemether/lumefantrine per tablet: Novartis, Switzerland) twice per day for 4 days at 0, 8, 24, 36, 48, 60, 72 and 84 h, each co-administered with 250 ml of chocolate milk containing 7 g of fat. All women were hospitalized during the treatment and antimalarial doses were directly supervised. The dose was repeated in full if vomiting occurred within 30 min after administration, or by a half dose if vomiting occurred between 30 and 60 min.

After the consent procedure and before drug administration, a full medical history including the previous history of malaria in the present pregnancy and examination (including obstetric evaluation and ultrasound) were carried out. Daily malaria smears were taken for microscopy until the woman became negative for asexual parasitaemia. During hospitalization, aural temperature, clinical and obstetric examinations, drug administration, and adverse events were assessed at least once daily. Thereafter women were seen weekly until delivery but for women who delivered before day 63, they completed the follow-up with post-partum visits i.e. there was a minimum follow-up period of 63 days. At delivery, malaria blood smears were prepared from the mother’s peripheral blood, cord blood, placental blood, and the neonate’s heel prick.

Recurrence of falciparum (or mixed infection) malaria parasites was treated with artesunate (2 mg/kg per day) and clindamycin (300 mg three times daily: Siam Bheasach, Thailand) for 7 days. Recurrence of vivax was treated with chloroquine (25 mg base/kg over 3 days, 250 mg tablet: Maneesh Pharmaceuticals, Ltd, India, and Medopharm, India) or artesunate alone or with clindamycin as above.

#### Laboratory procedures

Microscopy was used for detecting parasitaemia. Malaria parasites were counted per 1000 red blood cells (thin smear) or per 500 white blood cells (thick smear). Negative smears were declared after 200 high power fields (Obj X100, Ocular X10, FN: 18) were read on the thick blood smear. PCR was used only for assessing *P. falciparum* recrudescence: blood spots were collected on Whatman 3MM filter paper; DNA was extracted using QIAamp DNA mini kit and genotyped at three loci (*merozoite surface proteins 1* and *2* and *glutamate-rich protein*) to distinguish recrudescence (i.e. treatment failure) from novel infection (reinfection). As a post hoc analysis, mutations in *Pfkelch13* before treatment were assessed by nested PCR in those patients with *P. falciparum* mono-infections, and the copy number of *pfmdr1* was quantified by quantitative real-time PCR in the ASMQ and AL+ arms, as described previously [15, 16]. Haematocrit was read with a Hawksley scale on a capillary tube sample centrifuged at 10,000 rpm for 3 min. Computer-generated randomization scheduled a subgroup of 50 women in each drug arm to have full biochemistry and a complete blood count at baseline and on day 14 and 28. Biochemical analyses were performed at external accredited laboratories. Automated haematology analysers (a Sysmex pocH-100i until 2011 and a CeltacF MEK-8222 K by Nihon Kohden for the following years) were used. Electrocardiogram was assessed and reported elsewhere [17].

### Randomization, concealment of allocation and blinding

Study arms were allocated through a computer-generated randomization schedule (1:1:1 in blocks of 15). For the concealment of allocation, individual, sealed and sequentially numbered opaque envelopes kept at each trial site were used.

Administration of medication was open-label. Both patients and clinical staff knew the treatment given, but all laboratory staff reading the malaria smears and performing additional sample investigations (e.g. PCR genotyping) had no knowledge of the treatment received.

### Outcomes and definitions

The primary endpoint of the trial was the proportion of patients without recurrent malaria infection by delivery (or at least by day 63, whichever was longer). Recurrences could be recrudescences, reinfections, and for *P. vivax* relapses.

#### Falciparum malaria

For women who had falciparum malaria or co-infections of *P. falciparum* and *P. vivax* at enrolment, the efficacy endpoint was the PCR-corrected cure rate (S1 Table). PCR-confirmed recrudescent infections were classified as a treatment failure on the day of parasite reappearance [18]. Women who had recurrence with a PCR-confirmed novel *P. falciparum* infection (i.e. a new infection of *P. falciparum*) were censored on the day of reappearance [18]. Women with an indeterminate PCR or those who did not have PCR evaluated for the recurrence were excluded from the PCR-corrected efficacy analyses but included in PCR-uncorrected efficacy analyses [18]. PCR was conducted for all recurrent *P. falciparum* parasitaemia episodes even when women developed *P. vivax* parasitaemia before *P. falciparum* (intercalated episodes of vivax malaria). In this area, as *P. falciparum* is highly resistant to chloroquine [19] and chloroquine will neither cure nor prevent *P. falciparum* [20], they were not censored in the main analysis, but were censored on the day of detection of vivax parasitaemia in a sensitivity analysis.

#### Vivax malaria

For women who had vivax malaria or co-infections of *P. falciparum* and *P. vivax* at enrolment, the efficacy endpoint was recurrence-free survival. Recurrence of *P. vivax* was classified as treatment failure on the day of first recurrence of *P. vivax*. Women who developed *P. falciparum* parasitaemia before the recurrence of *P. vivax* parasitaemia were censored on the day when *P. falciparum* parasitaemia was detected.

#### Secondary endpoints

Further secondary efficacy endpoints included: time to fever and parasite clearance; and malaria parasite positivity at delivery of maternal and neonatal peripheral blood, placental blood, and cord blood. Fever was defined as a body temperature of ≥ 37.5 °C. Gametocyte carriage was measured as the number of weeks during which gametocytes were seen in the peripheral blood, divided by the total number of follow-up weeks, and expressed per 1000 person-weeks (person–gametocyte-weeks), in women without gametocytaemia at enrolment and without recurrence of falciparum malaria within 28(+ 3) days. Symptoms were screened for each day during treatment and at day 7 and day 14. Adverse events were classified into four groups from 1 (mild) to 4 (potentially life-threatening) as defined in the protocol using a standardized reference range for pregnant women [21]. Anaemia was defined as a haematocrit of less than 30%, and severe anaemia as a haematocrit below 20% [22].

Pregnancy outcomes included miscarriage (pregnancy loss prior to 28 weeks’ gestation); delivery which was reported as livebirth or stillbirth (prepartum or intrapartum); SGA defined as z-score ≤ 1.28 (10th percentile) using international standards [23] and low birth weight defined as < 2500 g in infants whose weight was measured in the first 72 h of life; preterm birth defined as delivery before 37 weeks’ gestation; and external congenital abnormality determined by a standardized newborn examination by trained health workers with confirmation by a trained physician. Low birth weight is presented only for historical reasons. Neonatal deaths were defined as live born infants who died within the first 28 days after birth.

### Statistical analyses

To calculate sample size requirements, the proportion of women without recurrence by delivery was estimated as AL^+^ 40%, ASMQ and DP 55%, with a ratio of 1:1 falciparum: vivax infections in the women recruited. A total of 1005 women (approximately 335 per arm) would then allow the detection of a significant difference in cure rates between AL^+^ and DP, assuming a two-sided alpha of 0.05, 90% power and 20% loss to follow-up.

Cumulative percentages at fixed time points of fever clearance (day 1, 2, 3), parasite clearance (day 1, 2, 3, 4, 5) and treatment efficacy (day 28, 42, 63, delivery) were estimated by the Kaplan-Meier estimator and the curves were compared using log-rank test or Wilcoxon test if not parallel. Multivariable Cox or linear regression was used for comparing different treatment arms and assessing the risk factors for four outcomes: *P. falciparum* recrudescence, haematocrit, *z*-score of birthweight and gestational age at birth. Variables were selected by backward elimination using a *p* value < 0.05 by likelihood ratio test as the cut-off. Treatment arms were included as a forced variable and DP was the reference standard to avoid multiple comparisons. For *P. falciparum* recrudescence, baseline parasitaemia was always included in the multivariable model as a known risk factor. The proportional hazards assumption was assessed by a global test. For assessing haematocrit, baseline haematocrit was always included in the model, but observations after blood transfusion and women who had recurrence of any malaria species within 63(+ 3) days were excluded. The proportions (and risk ratios) of adverse events (symptoms newly developed in the first 14 days) and other pregnancy outcomes among three treatment arms, and proportions of anaemia between falciparum and vivax malaria cases were compared by Fisher’s exact test (for categorical variables) or analysis of variance (for continuous variables). SPSS for Windows, version 11.0 and Stata MP 16.1 (Stata Corp, TX, USA) were used.

## Results

**Fig. 1:**
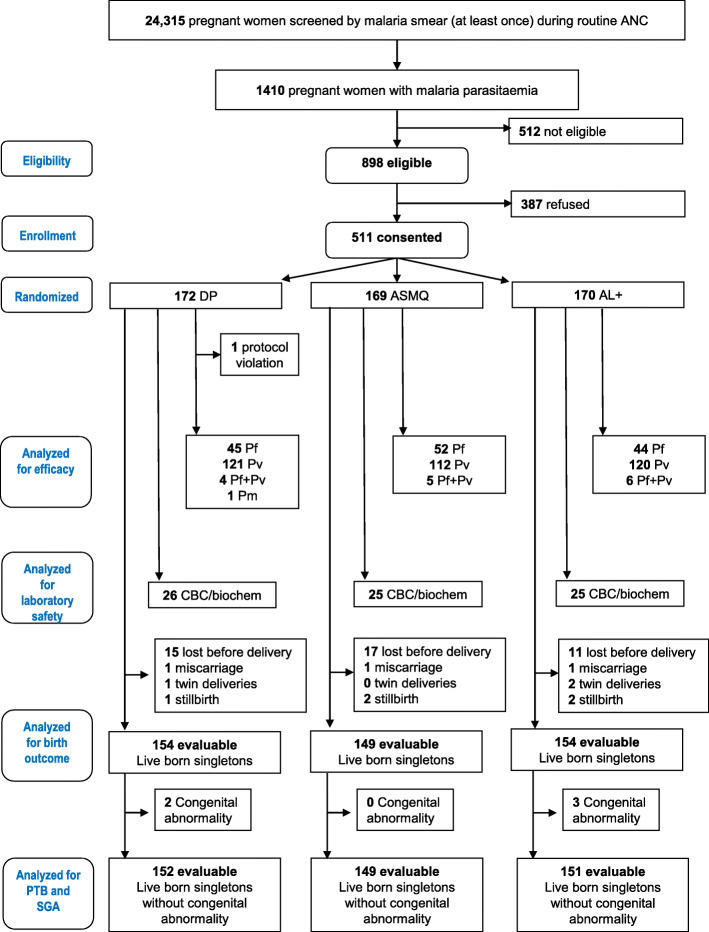
Participant flow in the randomized comparison of DP, ASMQ and AL^+^. Five hundred twelve did not meet eligibility criteria (underage *n* = 98, first trimester infection before amendment *n* = 155 or after amendment but no foetal heart beat visible *n* = 19, hyperparasitaemia *n* = 21, foetal demise at time of diagnosis *n* = 5, imminent labour *n* = 49, severe anaemia *n* = 3, very low parasitaemia *n* = 162). ANC: antenatal clinics, AL^+^: artemether-lumefantrine extended regimen, ASMQ: artesunate-mefloquine, CBC/biochem: complete blood count and biochemistry, DP: dihydroartemisinin-piperaquine, Pf: *Plasmodium falciparum*, Pm: *Plasmodium malariae*, PTB: preterm birth, Pv: *Plasmodium vivax*, SGA: small for gestational age

Between February 2010 and August 2016, 24,315 pregnant women were screened with malaria smears at least once and 1410 (5.8%) pregnant women were found positive for malaria parasites. Among them, 512 did not meet the eligibility criteria and 387 refused to participate, so 511 pregnant women participated in this trial (Fig. 1).

Successful elimination efforts in the region [24] impacted significantly on recruitment: from 106 recruited in the first year (2010) to 22 in the last year (2016) of the study (S1 Figure). The last *P. falciparum* episode was in 2015. As a result, enrolment was terminated due to futility in achieving the predetermined *P. falciparum* endpoint.

### Baseline characteristics

Of the 511 women with uncomplicated malaria who were enrolled, 142 (27.8%) had *P. falciparum,* 353 (69.1%) had *P. vivax*, 15 (2.9%) had both *P. falciparum* and *P. vivax*, and one woman had *Plasmodium malariae* (0.2%) infection. A minority of women (28.6%, 146/511) were febrile on admission, although most reported a history of fever (66.5%, 340/511). Of these 172 were randomized to receive DP, 169 to ASMQ, and 170 to AL^+^. ASMQ included 121 fixed and 48 loose dose regimens, with similar total doses (S2 Table). The baseline characteristics of the women in the three groups were similar overall (Table 1), and also when stratified by infecting malaria species (S3-5 Tables). Gestational age was estimated by ultrasound in 439 women (85.9%). After the amendment to the protocol allowing the first trimester women to be enrolled, 303 women were enrolled (59.3% of the total) of whom 15.2% (46/303) were in the first trimester of pregnancy. One woman enrolled in the DP group had *P. falciparum* hyperparasitaemia (4.6% infected red blood cells) and, as she did not fulfil the eligibility criteria, she was excluded from the efficacy analysis, but was included in the safety analyses.

**Table 1:** The baseline characteristics of enrolled women

Baseline characteristic	All (*n* = 511)	DP (*n* = 172)	ASMQ (*n* = 169)	AL^+^ (*n* = 170)
EGA at first ANC (week)^	15 [10–22]	15 [10–20]	15 [9–23.5]	15 [10–22.5]
EGA malaria (week)^	26 [19–33]	25 [19–32]	27 [19–34]	26 [19–32]
Trimester				
1st	46 (9.0%)	13 (7.6%)	15 (8.9%)	18 (10.6%)
2nd	236 (46.2%)	84 (48.8%)	74 (43.8%)	78 (45.9%)
3rd	229 (44.8%)	75 (43.6%)	80 (47.3%)	74 (43.5%)
Age (years)	25.7 (6.8)	25.7 (6.8)	26.3 (6.7)	25.1 (7.0)
Gravidity				
1	171 (33.5%)	60 (34.9%)	46 (27.2%)	65 (38.2%)
2	98 (19.2%)	30 (17.4%)	35 (20.7%)	33 (19.4%)
≥3	242 (47.4%)	82 (47.7%)	88 (52.1%)	72 (42.4%)
Parity				
0	204 (39.9%)	71 (41.3%)	57 (33.7%)	76 (44.7%)
1	97 (19.0%)	36 (20.9%)	32 (18.9%)	29 (17.1%)
≥2	210 (41.1%)	65 (37.8%)	80 (47.3%)	65 (38.2%)
Smoking	101 (19.8%)	32 (18.6%)	34 (20.1%)	35 (20.6%)
Height (cm)	151.0 (5.4)	151.2 (5.3)	151.1 (5.5)	150.8 (5.5)
Weight (kg)	51.7 (7.6)	52.4 (8.3)	52.0 (7.4)	50.8 (7.0)
Fever (temperature ≥ 37.5)	146 (28.6%)	45 (26.2%)	48 (28.4%)	53 (31.2%)
Fever (including history of fever)	340 (66.5%)	115 (66.9%)	113 (66.9%)	112 (65.9%)
Haematocrit (%)	32.1 (4.0)	32.1 (4.1)	32.3 (4.1)	32.0 (3.9)
Anaemia				
No anaemia	406 (79.5%)	138 (80.2%)	135 (79.9%)	133 (78.2%)
Moderate anaemia	105 (20.5%)	34 (19.8%)	34 (20.1%)	37 (21.8%)
Severe anaemia	0 (0.0%)	0 (0.0%)	0 (0.0%)	0 (0.0%)
First recorded malaria in pregnancy	277 (78.5%)	89 (73.6%)	94 (83.9%)	94 (78.3%)
Species				
Pf mono-infection	142 (27.8%)	46 (26.7%)	52 (30.8%)	44 (25.9%)
Pv mono-infection	353 (69.1%)	121 (70.3%)	112 (66.3%)	120 (70.6%)
Pf and Pv coinfection	15 (2.9%)	4 (2.3%)	5 (3.0%)	6 (3.5%)
Pm mono-infection	1 (0.2%)	1 (0.6%)	0 (0.0%)	0 (0.0%)
Pf parasitaemia (/uL)*	5689 (16–207994)	4561 (16–207994)	5805 (16–124595)	6936 (96–120199)
Pv parasitaemia (/uL)*	658 (16–83524)	674 (16–83524)	651 (16–29893)	648 (16–40694)
Presence of Pf gametocytes	24 (4.7%)	12 (7.0%)	6 (3.6%)	6 (3.5%)
Presence of Pv gametocytes	233 (45.6%)	81 (47.1%)	66 (39.1%)	86 (50.6%)

*AL*^*+*^ artemether-lumefantrine extended regimen, *ANC* antenatal care, *ASMQ* artesunate-mefloquine, *BMI* body mass index, *DP* dihydroartemisinin-piperaquine, *EGA* estimated gestational age, *Pf Plasmodium falciparum*, *Pm Plasmodium malariae*, *Pv Plasmodium vivax*, *SD* standard deviation. Data are presented as mean (standard deviation), number (%), ^median [interquartile range] or *geometric mean (range)

Anaemia was defined as a haematocrit of 20-29% (moderate) or less than 20% (severe)

### Efficacy endpoints

The primary outcome, the recurrence of parasitaemia of any malaria species, was assessed in 510 women (Table 2). Recurrence was more frequent in the AL^+^ group than in the other two groups until around 90 days (S2 Figure, Panel a). The Kaplan-Meier cumulative proportion of women without recurrences by delivery was 37.3% (95% confidence interval [CI] 18.7 to 56.0) for DP, 48.7% (95% CI 37.3 to 59.2) for ASMQ, and 43.7% (95% CI 26.5 to 59.7) for AL^+^: the sum of ranks was lowest in DP, followed by ASMQ and then AL^+^ (*p* = 0.03 by Wilcoxon test). There was no difference in proportions between fixed dose and loose dose ASMQ (*p*=0.74).

**Table 2:** Cumulative proportions of treatment success (adequate clinical and parasitological response) for each treatment arm at fixed time points in pregnant women

	Cumulative percentage of treatment success (95% CI) estimated by Kaplan-Meier method			*p* value
	DP	ASMQ	AL^+^	
P. falciparum* PCR-corrected	*N* = 49	*N* = 55	*N* = 50	0.13
Day 28	93.7% (81.6–97.9)	81.5% (68.4–89.6)	91.9% (79.8–96.9)	
Day 42	93.7% (81.6–97.9)	81.5% (68.4–89.6)	87.5% (74.3–94.2)	
Day 63	93.7% (81.6–97.9)	79.6% (66.1–88.1)	87.5% (74.3–94.2)	
Delivery	93.7% (81.6–97.9)	79.6% (66.1–88.1)	87.5% (74.3–94.2)	
P. falciparum* PCR-uncorrected	*N* = 49	*N* = 57	*N* = 50	0.07
Day 28	93.7% (81.6–97.9)	80.4% (67.4–88.6)	91.9% (79.8–96.9)	
Day 42	91.4% (78.7–96.7)	80.4% (67.4–88.6)	87.5% (74.3–94.2)	
Day 63	91.4% (78.7–96.7)	78.5% (65.3–87.2)	87.5% (74.3–94.2)	
Delivery	91.4% (78.7–96.7)	70.7% (53.8–82.4)	84.6% (70.2–92.4)	
P. vivax*	*N* = 125	*N* = 117	*N* = 126	0.0006^‡^
Day 28	100.0%	100.0%	95.8% (90.2–98.2)	
Day 42	98.3% (93.4–99.6)	99.0% (93.2–99.9)	79.3% (70.8–85.6)	
Day 63	82.9% (74.5–88.8)	89.5% (81.4–94.2)	68.5% (59.0–76.1)	
Delivery	20.6% (5.1–43.4)	46.0% (30.9–60.0)	28.7% (10.0–50.8)	
Any malaria recurrence	*N* = 171	*N* = 169	*N* = 170	0.03^‡^
Day 28	98.2% (94.6–99.4)	92.5% (87.2–95.7)	93.9% (88.9–96.6)	
Day 42	95.1% (90.4–97.5)	90.6% (84.8–94.2)	79.2% (72.0–84.7)	
Day 63	83.1% (76.1–88.2)	80.8% (73.4–86.2)	69.9% (62.0–76.4)	
Delivery	37.3% (18.7–56.0)	48.7% (37.3–59.2)	43.7% (26.5–59.7)	

*AL*^*+*^ artemether-lumefantrine extended regimen, *ASMQ* artesunate-mefloquine, *CI* confidence interval, *DP* dihydroartemisinin-piperaquine, *PCR* polymerase chain reaction

*Including co-infection of *P. falciparum* and *P. vivax*

*p* values by log-rank test or Wilcoxon test (^‡^). For all endpoints, + 3 days allowed

There were no differences in fever and parasite clearance times among the ACTs evaluated (S6 Table) although clearance times were slower in falciparum compared to vivax malaria (S6 Table, S3 Figure). Gametocyte carriage, placental and congenital malaria were uncommon, and are detailed in the supplement (S7 Table).

#### Falciparum malaria

**Fig. 2:**
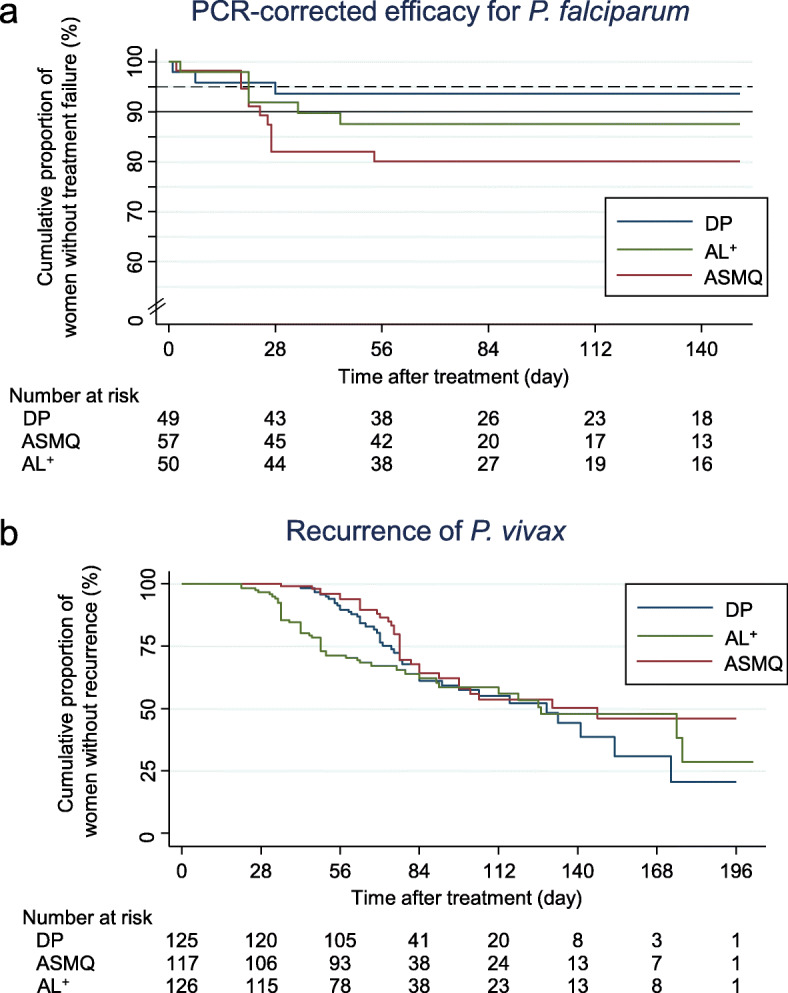
Cumulative proportions of treatment success over time for each arm stratified by malaria species estimated by Kaplan-Meier estimator. **a** *Plasmodium falciparum* PCR-corrected treatment success. **b** *Plasmodium vivax* treatment success. Black dotted line shows 95% and solid line 90%, which is the efficacy threshold set by the World Health Organization to replace the treatment [3]

In the 156 women with *P. falciparum* infection (including 15 with *P. vivax* co-infection), only DP (93.7%, 95% CI 81.6 to 97.9) gave a > 90% PCR-corrected cure rate by delivery. This compared with 79.6% (66.1 to 88.1) for ASMQ and 87.5% (74.3 to 94.2) for AL^+^ (Table 2, Fig. 2a). This difference was not significant (*p*=0.13). Early treatment failure occurred in three women (one in each treatment arm). PCR-confirmed recrudescence occurred in 17 women at a median interval of 24 days (range 7–54): two in DP, ten in ASMQ and five in AL^+^. A sensitivity analysis censoring intercalated vivax infection gave results similar to the main analysis (S2 Figure, panel b). PCR was not available in two ASMQ recurrences. There were only three re-infections (one in each treatment arm), so the PCR-uncorrected efficacy was similar to the PCR-corrected efficacy (S2 Figure, panel c).

Both the unadjusted and adjusted risks of treatment failure after uncomplicated falciparum malaria were higher in ASMQ group (adjusted hazard ratio [aHR] 3.00, 95% CI 0.84 to 10.77, *p*=0.09) and in the AL^+^ group (aHR 1.85, 95% CI 0.46 to 7.40, *p*=0.39) than DP, although the difference was not significant (S8 Table).

Vivax malaria after *P. falciparum* mono-infection was common (S2 Figure, panel d): 24.0% (95% CI 16.8 to 33.6) by day 120. The median (range) intervals to recurrence were 84 (35–152) days after DP (*n*=9), 73 (49–144) after ASMQ (*n*=10) and 49 (21–80) after AL^+^ (*n*=12).

#### Molecular markers of antimalarial resistance

Propeller mutations in *Pfkelch13* were assessed in 130 *P. falciparum* mono-infection samples (92%, 130/141) before treatment. The overall prevalence of any *Pfkelch13* propeller mutations was 65% (85/130). This was slightly but not significantly lower in ASMQ group: DP (70%, 30/43), ASMQ (56%, 25/45) and AL^+^ (71%, 30/42). Treatment failure was almost exclusively observed (95%, 18/19) in those infections with *Pfkelch13* mutations; 2% (1/45) in infections with wild type but 21% (18/86) in infections with *Pfkelch13* mutations; risk ratio (RR) 9.42 (95% CI 1.30 to 68.29). When the presence of *Pfkelch13* mutations was added in the Cox model for the PCR-corrected efficacy, the estimates were similar to those obtained by the model without *Pfkelch13* mutations (S9 Table). Slow parasite clearance was associated with *Pfkelch13* mutations: 95% (36/38) of women who remained parasitaemic on day 3 had infections with *Pfkelch13* mutations. The prevalence of *Pfkelch13* mutations increased steadily over time: 44% (12/27) in 2010, 51% (19/37) in 2011, 81% (25/31) in 2012 and 87% (26/30) in 2013.

The copy number of *pfmdr1* was assessed in 98 samples in ASMQ or AL^+^ groups (92%, 98/107). The prevalence of *P. falciparum* parasites harbouring multiple copies of *pfmdr1* was 47% (46/98): 23 had two copies, 22 had three, and one had four. The risk of failure was higher in infections with multiple copies of *pfmdr1* (35%, 16/46) compared with those with single copy (2%, 1/52); RR 18.09 (95% CI 2.49 to 131.12). In ASMQ and AL^+^ groups, infections with *Pfkelch13* mutations (aHR 9.50, 95% CI 1.24 to 72.83, *p*=0.03) and multiple copies of *pfmdr1* (aHR 17.81, 95% CI 2.30 to 137.99, *p*=0.0006) were independently associated with higher risks of treatment failure (data not shown). There was no temporal trend for *pfmdr1* copy number.

Only one woman had more than one recrudescence. After the initial treatment with ASMQ, three recrudescences were treated with artesunate plus clindamycin for seven days except the second recrudescence stopped after one day as the participant left the clinic against medical advice. The patient’s infection had a *Pfkelch13* mutation (R561H) and three copies of *pfmdr1*.

#### Vivax malaria

In the 368 women presenting with *P. vivax* infection (including 15 with *P. falciparum* co-infections), the median time to the first recurrence was 63 days (range 21–177). Both DP (43 recurrences, median 70 days) and ASMQ (32 recurrences, median 76 days,) gave longer recurrence-free periods than AL^+^ (48 recurrences, median 45.5 days) (Table 2, Fig. 2b). By delivery, recurrence-free proportions had fallen to 20.6% (95% CI 5.1 to 43.4) for DP, 46.0% (30.9 to 60.0) for ASMQ and 28.7% (10.0 to 50.8) for AL^+^. The three curves were different among treatments: the sum of ranks was lowest in ASMQ, followed by DP and then AL^+^ (*p*=0.0006 by Wilcoxon test).

### Anaemia

Overall, 20.5% (105/511) of women were anaemic (haematocrit < 30%) at baseline (Table 1): more with falciparum malaria (31.5%, 45/142) compared to vivax malaria (16.1%, 57/353, *p*=0.0001) or mixed infections (20.0%, 3/15, *p*=0.56). Eight women (1.6%) were transfused (two in DP, and three each in ASMQ and AL^+^) for malaria-related anaemia. Six women received blood transfusions within 10 days of treatment, five (3.5%, 5/142) after falciparum and one (0.3%, 1/353) after vivax malaria. Two other women received after 4 weeks relating to falciparum recurrence.

The haematocrits of women with falciparum malaria were lower than those of women with vivax malaria on days 3, 7 and 28 and lowest on day 3. Haematocrits had recovered back to or above pre-treatment levels by week 3 following *P. vivax* infections and by week 5 following *P. falciparum* infections (S4 Figure, panel a); with no significant difference among the treatments (S4 figure, panel b) and also in the analysis adjusting for baseline characteristics (data not shown).

### Serious adverse events

One unexpected maternal death occurred on the day after completing treatment with ASMQ in a patient with vivax malaria who developed acute respiratory distress syndrome (presumed pulmonary oedema) unresponsive to mechanical ventilation. The foetus was stillborn. This case is detailed elsewhere [25].

### Other adverse events

The reported symptoms before treatment were comparable among the three arms (S10 Table). After treatment, women treated with ASMQ complained more of nausea (13.7%, 16/117, RR 5.01, 95% CI 1.50 to 16.74), vomiting (13.4%, 18/134, RR 9.40, 95% CI 2.22 to 39.75), dizziness (37.1%, 26/70, RR 3.45, 95% CI 1.61 to 7.40) and sleep disturbance (14.0%, 15/107, RR 1.78, 95% CI 1.35 to 2.36) compared with DP and AL^+^ combined, which were generally very well tolerated. The risks were not different between two formulations of ASMQ in any of the symptoms (data not shown). All these symptoms were mild and transient. The proportions of women with abnormal biochemistry and complete blood count measurements over time were small after treatment (i.e. day 7 and 14) and not different among different treatment arms (data not shown). One woman had a grade 3 adverse event (hyponatraemia at 124 mmol/L on day 28) in the AL^+^ group. There were no grade 4 adverse events.

### Birth outcomes

Among the 511 pregnant women enrolled, 43 (8.7%, 15/172 in DP; 10.1%, 17/169 in ASMQ; and 6.5%, 11/170 in AL^+^) were lost to follow up before delivery and so pregnancy outcomes were unknown (Table 3). The numbers of miscarriages (including women treated in the first trimester), preterm births, stillbirths, congenital abnormalities and neonatal deaths were low and there was no difference in the incidence of these adverse birth outcomes among the different treatment arms (Table 3, S11-S15 Table). Most normal live born singletons were weighed within 72 h of birth (88%, 397/452), and 26.2% (104/397) were SGA.

**Table 3:** Summary of the pregnancy outcomes for each treatment group

Baseline characteristic	DP	ASMQ	AL^+^	*p* value
Malaria				
Malaria before 28 weeks EGA	97/172 (56%)	89/169 (53%)	96/170 (56%)	
Malaria before 37 weeks EGA	165/172 (96%)	153/169 (91%)	162/170 (95%)	
Follow-up				
Followed up until ≥ 28 weeks	91/97 (94%)	85/89 (96%)	92/96 (96%)	
Followed up until delivery	157/172 (91%)	152/169 (90%)	159/170 (94%)	
Birth outcomes				
Twins	1/157 (1%)	0/152 (0%)	2/159 (1%)	
Miscarriage	1/91 (1%)	1/85 (1%)	1/90 (1%)	1.00
Stillbirth	1/155 (1%)	2/151 (1%)	2/156 (1%)	0.87
Congenital abnormality	2/155 (1%)	0/151 (0%)	3/156 (2%)	0.38
Male	70/155 (45%)	75/151 (50%)	75/155 (48%)	0.73
EGA (week)*	38.8 (1.9)	39.0 (2.0)	38.8 (2.1)	0.56
Preterm birth*	13/145 (9%)	8/135 (6%)	14/143 (10%)	0.45
Birthweight weighed within 3 days*	134/152 (88%)	130/148 (88%)	136/150 (91%)	
Birthweight (g)*^‡^	2860 (493)	2926 (396)	2881 (524)	0.51
Small for gestational age*^‡^	32/133 (24%)	34/129 (26%)	38/135 (28%)	0.76
Low birthweight*^‡^	28/134 (21%)	14/130 (11%)	26/136 (19%)	0.06
Height (cm)*	48.8 (2.7)	49.3 (2.6)	49.0 (3.3)	0.48
Arm circumference (cm)*	10.4 (1.1)	10.5 (0.9)	10.3 (1.2)	0.47
Head circumference (cm)*	32.0 (1.6)	32.2 (1.7)	31.9 (1.8)	0.41
Apgar 5 min*				
0–3	0/115 (0%)	1/116 (1%)	3/125 (2%)	0.17
4–6	1/115 (1%)	0/116 (0%)	3/125 (2%)	
7–10	114/115 (99%)	115/116 (99%)	119/125 (95%)	
Placental weight (g)*	508 (111)	510 (95)	510 (121)	0.98

*AL*^*+*^ artemether-lumefantrine extended regimen, *ASMQ* artesunate-mefloquine, *BMI* body mass index, *DP* dihydroartemisinin-piperaquine, *EGA* estimated gestational age, *Pf Plasmodium falciparum*, *Pm Plasmodium malariae*, *Pv Plasmodium vivax*. *P* value by Fisher’s exact test or ANOVA test. Figures are shown in mean (standard deviation) or number (%).

*Include only live singleton births without congenital abnormality

^‡^Include only those who were weighted within 3 days

Factors associated with SGA were assessed by univariable and multivariable linear regression using SGA *z*-score as the outcome (S16 Table). Treatment was not associated with SGA *z*-score (*p* = 0.63) while there was an inverse linear association with the number of malaria episodes during pregnancy (− 0.14/episode, 95% CI − 0.22 to − 0.07, *p*=0.0001). The SGA *z*-score was also lower in pregnant women with moderate anaemia at the time of the malaria episode (− 0.24, 95% CI − 0.45 to − 0.03, *p*=0.03) compared with those without anaemia. The SGA *z*-score was higher with taller maternal height (0.03/cm, 95% CI 0.02 to 0.05, *p*< 0.0001) and higher maternal body mass index (0.05/kg/m^2^, 95% CI 0.02 to 0.08, *p*=0.0005), but lower in primigravid (gravidity = 1) women (− 0.35, 95% CI − 0.54 to − 0.17, *p*=0.0002) compared with multigravid (gravidity ≥ 3) women. Birth outcomes in pregnant women who had malaria in the first trimester and followed up until delivery (41/46) were similar to the outcomes in all participants, including length and weight of the infant at birth (S17 Table).

## Discussion

The rapid rise in artemisinin-resistant falciparum malaria over the past 13 years in the GMS has compromised the treatment of falciparum malaria in the affected areas. Pregnant women are an important and vulnerable group in whom antimalarial pharmacokinetics are altered, treatment responses are often impaired and adverse outcomes are common for both the mother and the foetus. In this RCT comparing three currently available ACTs for women with malaria detected during antenatal clinic follow-up, treatment efficacy was highest for DP for falciparum malaria despite the increased prevalence of *Pfkelch13* mutations. This presumably reflects the continued efficacy of piperaquine in the Western GMS. DP has also proved a well-tolerated and safe treatment as well as prevention in pregnant women in sub-Saharan African countries [26–30]. In contrast to the Eastern GMS where piperaquine resistance has emerged and compromised DP efficacy, elsewhere this combination retains excellent efficacy. At the same time, whereas ASMQ is currently highly efficacious in the Eastern GMS, it is less efficacious on the Thailand-Myanmar border where mefloquine resistance is prevalent. The threefold increased adjusted risk (aHR 3.00, 95% CI 0.84 to 10.77) of *P. falciparum* failure after ASMQ compared to DP, although not statistically significant (*p* = 0.09), is consistent with contemporary efficacy studies in larger non-pregnant populations [16, 31]. Treatment failure after ASMQ or AL is known to be associated with an increased copy number of *pfmdr1* [15]. In this study, treatment failure in all three treatment arms occurred almost exclusively (95% of all recrudescences) in *P. falciparum* infections with *Pfkelch13* mutations, and increased copy number of *pfmdr1* was an independent risk factor for failure after ASMQ or AL^+^ treatment. This emphasises the importance of artemisinin resistance as a major determinant of ACT treatment failure and suggests that molecular markers of piperaquine resistance and DP clinical efficacy will need close monitoring in the near future. Realizing that falciparum malaria could potentially become untreatable, intense malaria elimination activities have been conducted in this previously highly malarious region along the Thailand-Myanmar border [24]. These have been very successful. Indeed, the incidence of falciparum malaria has fallen so dramatically in the region that this RCT in pregnant women was forced to stop for lack of cases (S1 Figure).

The efficacy of the extended AL^+^ regimen of five tablets twice a day for 4 days of 87.6% (95% CI 74.4 to 94.2) was only slightly better than the performance of the standard AL regimen [cure rate 82.0% (74.8 to 89.3)] in pregnant women in this area between 2004 and 2006 [12]. However, a careful interpretation is needed: during the earlier study, AL efficacy was not compromised by artemisinin resistance as the prevalence of *Pfkelch13* propeller mutations was very low at that time [16]. The 5-day AL regimen for pregnant women evaluated recently in the Democratic Republic of the Congo [32] would have been preferable providing greater parasite reduction by exposing three successive asexual cycles to artemether, but the more rapidly eliminated lumefantrine with its shorter post-treatment prophylactic effect remains a disadvantage for pregnant women in endemic areas compared with other partner drugs that have longer half-lives.

This study reports the largest number of *P. vivax* infections as well as women in the first trimester of pregnancy to be included in an RCT in pregnant women. One earlier small RCT in Columbia included 20 pregnant women in their first trimester and compared efficacy and tolerability, but not pregnancy outcomes, of chloroquine and amodiaquine [33]. There have been no previous comparative trials of ACTs in *P. vivax* malaria in pregnancy. As radical cure with primaquine cannot be given in pregnancy, the efficacy reported here is a composite of blood stage cure and relapse suppression. *P. vivax* recurrence was very common in this study, as in Colombia where 30% of women experienced recurrences within 120 days after chloroquine treatment [34]. Recurrent vivax malaria was by far the main complication of malaria in pregnancy. After falciparum malaria, *P. vivax* occurred in approximately one quarter of women within 4 months, and it was more common than *P. falciparum* recrudescence. In vivax malaria, recurrence of *P. vivax* occurred in approximately two thirds of women. Subsequent recurrences of *P. vivax* were suppressed by the slowly eliminated antimalarials, with mefloquine slightly outperforming piperaquine and, as expected, lumefantrine [35]. Most, if not all, of these recurrences were likely to be relapses.

There were no surprises in the comparative tolerability evaluations. ASMQ was less well tolerated than AL^+^ or DP with higher proportions of nausea, vomiting, and sleep disturbance [4, 26, 36]. The incidence of adverse birth outcomes was low and similar among the three ACTs. While small, this randomized series with intentional first trimester ACT use and no untoward outcomes concurs with previous reports from Asia and Africa [5, 8]. ACTs are more efficacious, effective and better tolerated than quinine in the treatment of malaria. The continued WHO Global Malaria Programme recommendation for quinine treatment of uncomplicated falciparum malaria in the first trimester is unjustified and unwarranted, and should have been changed years ago (as recommended by the WHO expert review group) [6, 7]. The prevalence of SGA (26.3%) was slightly higher than the regional estimate (21.6%, 14.2 to 37.7) [37], but similar to that among women who had malaria in pregnancy in this area reported previously (27%) [38]. The positive association between the increased number of malaria episodes in pregnancy and SGA is consistent with previous reports [9, 38]. This emphasizes the importance of prevention of malaria in pregnancy. This can be achieved temporarily by partner drugs with longer post-treatment prophylaxis, or more reliably by providing weekly vivax chemoprophylaxis with chloroquine beginning on day 21 after the primary episode (including both *P. falciparum* and *P. vivax*). This has been implemented as a consequence of this trial.

Limitations of this study include termination of the RCT before the sample size was reached and loss-to-follow-up before delivery (8.4%), both of which reduced the statistical power of the study. Chloroquine was not included partly because of concerns over chloroquine-resistance *P. vivax* in this area [39] although it remains first-line treatment. WHO recommends ACT as the first-line treatment for both falciparum and vivax malaria, even in the area with chloroquine-susceptible vivax [3], which is operationally easier. Estimation of gestational age is critical to assess preterm birth and SGA. In this cohort, while all women had at least one ultrasound assessment of gestation with a median gestation of the first scan at 15 (interquartile range 10–22 weeks), 86% had final gestation determined by ultrasound (14% of late presenters by Dubowitz), ensuring the quality of the assessment. Finally, this trial is open-label. Although some subjective outcomes, such as the tolerability, could have been biased by previous experience, this seems unlikely; AL^+^ had never been tested in this area, DP was not in use in pregnancy, the observed effects such as vomiting are objective and the findings from this trial are consistent with tolerability from pregnancy treatment studies elsewhere [4, 26].

## Conclusions

In this RCT of three ACTs conducted on the Thailand-Myanmar border during a period of increasing artemisinin resistance, DP was the only ACT providing greater than 90% cure of falciparum malaria. On the other hand, ASMQ provided the longest post-treatment suppression of *P. vivax* recurrence but was less tolerated than DP or AL^+^. Identification of species is thus important for selecting the first-line drug. The main cause of recurrent malaria for all infections was *P. vivax* relapse. Prevention of vivax malaria recurrence requires suppressive prophylaxis with chloroquine. Hence treatment on the Thailand-Myanmar border for uncomplicated malaria is currently the same for pregnant and non-pregnant patients: DP for falciparum and chloroquine for vivax malaria. Importantly, because primaquine radical cure cannot be given, chloroquine prophylaxis is now given from the third week after treatment until term. For the fetus, all three ACTs appeared safe as treatment including in the first trimester.

## Supplementary Information

**Additional file 1: S1 Table.** Definition of primary efficacy endpoint by initial species. **S2 Table.** Dose of each compound given to the participants. **S3 Table.** The baseline characteristics of women with *Plasmodium falciparum* mono-infection. **S4 Table.** The baseline characteristics of women with *Plasmodium vivax* mono-infection. **S5 Table.** The baseline characteristics of patients with *Plasmodium falciparum* infection, including both *P. falciparum* mono-infection and co-infection of *P. falciparum* and *Plasmodium vivax*. **S6 Table.** Cumulative proportion of fever and parasite clearance of falciparum and vivax for each treatment arm among pregnant women with history of fever or documented fever at enrolment. **S7 Table**. Description of gametocyte carriage, congenital and placental malaria. **S8 Table.** Univariable and multivariable analyses of the risk of PCR-corrected treatment failure in pregnant women with uncomplicated falciparum malaria using a Cox proportional hazard model. **S9 Table.** Univariable and multivariable analyses on the risk of PCR-corrected treatment failure in pregnant women with uncomplicated falciparum malaria using a Cox proportional hazard model with *Pfkelch13* mutations. **S10 Table.** Prevalence of symptoms before and after treatment. **S11 Table.** Description of pregnancy outcomes. **S12 Table.** Details of pregnant women who had miscarriage or stillbirth. **S13 Table.** Details of congenital abnormality and ICD-10 coding. **S14 Table.** Details of neonatal mortality. **S15 Table.** Univariable and multivariable linear regression analyses of the characteristics associated with gestational week at birth among those who had malaria in pregnancy. **S16 Table.** Univariable and multivariable linear regression analyses of the characteristics associated with SGA z-score (birthweight for gestational age and newborn sex at birth). **S17 Table.** Birth outcomes in pregnant women who had malaria and were enrolled in the first trimester. **S1 Figure.** Monthly number of pregnant women with malaria for each malaria species over the study period (from February 2010 to August 2016). **S2 Figure.** Kaplan-Meier survival curves for antimalarials in pregnancy: Panel a. recurrence of any malaria species, Panel b. *Plasmodium falciparum* PCR-corrected efficacy censoring intercalated *Plasmodium vivax* malaria, Panel c. *P. falciparum* PCR-uncorrected efficacy , and Panel d. *P. vivax* parasitaemia after *P. falciparum* mono-infection. **S3 Figure.** Kaplan-Meier survival curves for parasite clearance of *Plasmodium falciparum* (Panel a) or *Plasmodium vivax* (Panel b) for each treatment arm. **S4 Figure.** Fractional change in haematocrit from baseline during the follow-up for each species (Panel a) or treatment stratified by malaria species (Panel b).

## Data Availability

Data are available from MORU Tropical Health Network upon request from the link below. (https://www.tropmedres.ac/units/moru-bangkok/bioethics-engagement/data-sharing)

## Abbreviations

ACT: Artemisinin-based combination therapy
aHR: Adjusted hazard ratio
AL^+^: Extended artemether-lumefantrine regimen
ANC: Antenatal clinic
ASMQ: Artesunate-mefloquine
CI: Confidence interval
DP: Dihydroartemisinin-piperaquine
GMS: Greater Mekong Subregion
HR: Hazard ratio
PCR: Polymerase chain reaction
Pf: *Plasmodium falciparum*
Pv: *Plasmodium vivax*
RCT: Randomized controlled trial
RR: Risk ratio
SD: Standard deviation
SGA: Small for gestational age
WHO: The World Health Organization
