# Supplementary material for: A randomized controlled trial of dihydroartemisinin-piperaquine, artesunate-mefloquine and extended artemether-lumefantrine treatments for malaria in pregnancy on the Thailand-Myanmar border
Source: BMC Med. 2021 Jun 10;19:132. doi: 10.1186/s12916-021-02002-8 (PMC8191049; doi:10.1186/s12916-021-02002-8)
Supplement: Supplementary file 1 — Additional file 1: S1 Table. Definition of primary efficacy endpoint by initial species. S2 Table. Dose of each compound given to the participants. S3 Table. The baseline characteristics of women with Plasmodium falciparum mono-infection. S4 Table. The baseline characteristics of women with Plasmodium vivax mono-infection. S5 Table. The baseline characteristics of patients with Plasmodium falciparum infection, including both P. falciparum mono-infection and co-infection of P. falciparum and Plasmodium vivax. S6 Table. Cumulative proportion of fever and parasite clearance of falciparum and vivax for each treatment arm among pregnant women with history of fever or documented fever at enrolment. S7 Table. Description of gametocyte carriage, congenital and placental malaria. S8 Table. Univariable and multivariable analyses of the risk of PCR-corrected treatment failure in pregnant women with uncomplicated falciparum malaria using a Cox proportional hazard model. S9 Table. Univariable and multivariable analyses on the risk of PCR-corrected treatment failure in pregnant women with uncomplicated falciparum malaria using a Cox proportional hazard model with Pfkelch13 mutations. S10 Table. Prevalence of symptoms before and after treatment. S11 Table. Description of pregnancy outcomes. S12 Table. Details of pregnant women who had miscarriage or stillbirth. S13 Table. Details of congenital abnormality and ICD-10 coding. S14 Table. Details of neonatal mortality. S15 Table. Univariable and multivariable linear regression analyses of the characteristics associated with gestational week at birth among those who had malaria in pregnancy. S16 Table. Univariable and multivariable linear regression analyses of the characteristics associated with SGA z-score (birthweight for gestational age and newborn sex at birth). S17 Table. Birth outcomes in pregnant women who had malaria and were enrolled in the first trimester. S1 Figure. Monthly number of pregnant women with malaria for each mal [file 12916_2021_2002_MOESM1_ESM.pdf]

A randomized controlled trial of dihydroartemisinin-piperaquine, artesunate-mefloquine and extended artemether-lumefantrine treatments for malaria in pregnancy on the Thailand-Myanmar border

Supplementary File

S1 Table. Definition of primary efficacy endpoint by initial species

Initial species	First recurrence	Pf efficacy - Delivery or day 63 whichever occurred later	Pv efficacy - Delivery or day 63 whichever occurred later
Pf	No	ACPR	-
Pf	Pf	Fail (PCR needed)	-
Pf	Pv	Not censored	-
Pv	No	-	ACPR
Pv	Pf	-	Censored
Pv	Pv	-	Fail
Mixed (Pf+Pv)	No	ACPR	ACPR
Mixed (Pf+Pv)	Pf	Fail (PCR needed)	Censored
Mixed (Pf+Pv)	Pv	Not censored	Fail
Pm	Any	-	-

ACPR: adequate clinical and parasitological response (treatment success), EGA: estimated gestational age, PCR: polymerase chain reaction, Pf: *Plasmodium falciparum*, Pm: *Plasmodium malariae*, Pv: *Plasmodium vivax*.

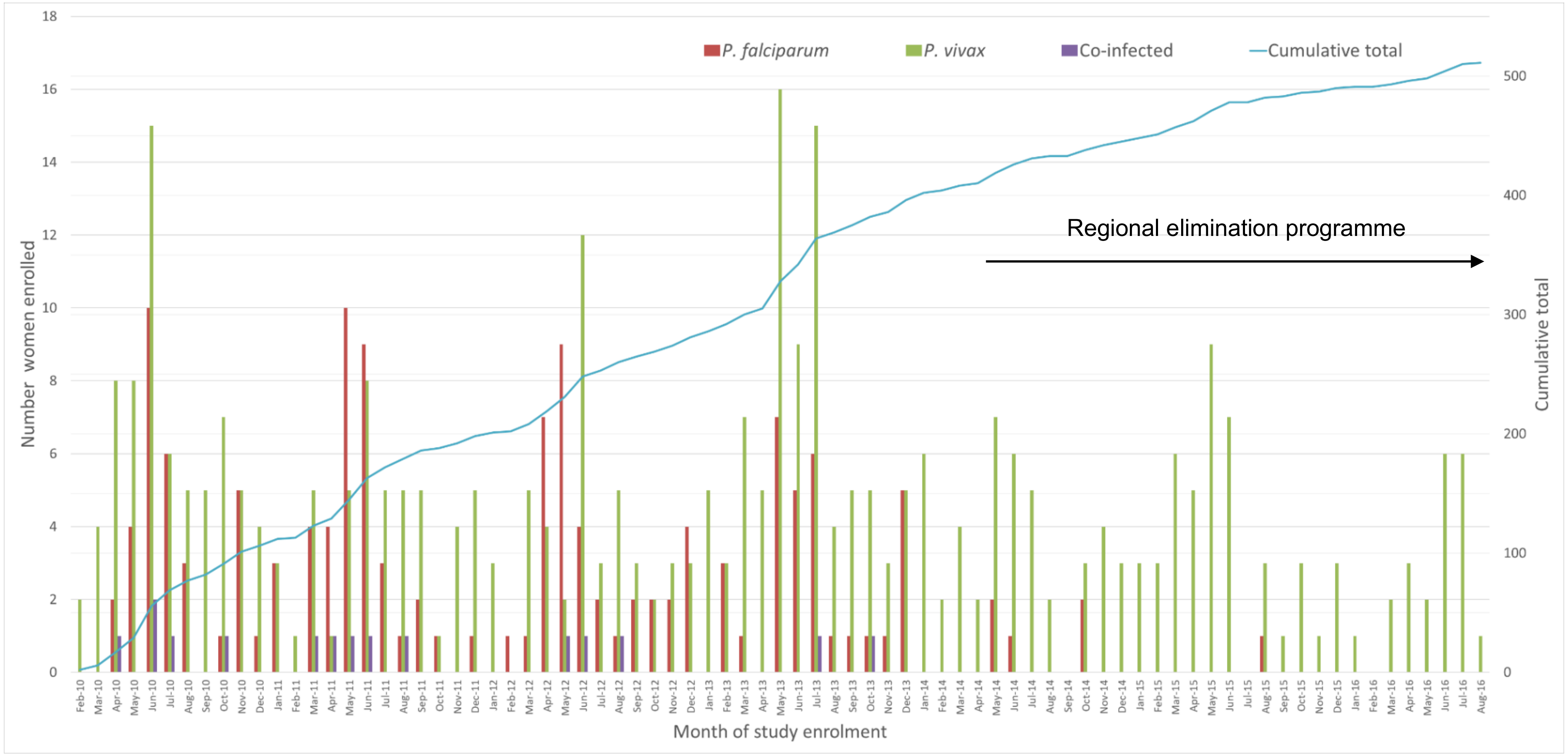

Footnote: Single *P. malariae* in Jul-2011 included as vivax for this figure only.

S1 Figure. Monthly number of pregnant women with malaria enrolled in the study for each malaria species over the study period February 2010 to August 2016

S2 Table. Dose of each compound given to the participants

Treatment	N	Mean (range) of total dose (mg/kg body weight)
DP*	172	
Dihydroartemisinin		6·5 (4·7-7·3)
Piperaquine		52·0 (37·6-58·5)
ASMQ	169	
Artesunate		11·8 (9·0-15·8)
Mefloquine		25·2 (19·7-34·7)
ASMQ – fixed dose	121	
Artesunate		11·7 (9·0-15·8)
Mefloquine		25·8 (19·7-34·7)
ASMQ – loose dose	48	
Artesunate		12·0 (11·5-12·3)
Mefloquine		23·5 (21·6-25·6)
AL+	170	
Artemether		16·1 (11·1-24·2)
Lumefantrine		96·3 (66·7-145·5)

AL+: artemether-lumefantrine extended regimen, ASMQ: artesunate-mefloquine, DP: dihydroartemisinin-piperaquine.
* Including one patient with protocol violation (i.e. hyperparasitaemia).

S3 Table. The baseline characteristics of patients with *Plasmodium falciparum* mono-infection

Baseline characteristic	Mean (SD), Percentage (n), Median (range)			
	All (n=142)	DP (n=46)	ASMQ (n=52)	AL+ (n=44)
EGA (week)	24·7 (8·4)	23·6 (8·2)	25·5 (8·8)	24·8 (8·2)
Trimester				
1st	11·3 (16)	10·9 (5)	11·5 (6)	11·4 (5)
2nd	50·0 (71)	54·3 (25)	42·3 (22)	54·5 (24)
3rd	38·7 (55)	34·8 (16)	46·2 (24)	34·1 (15)
Age (years)	26·1 (7·3)	25·7 (7·6)	27·0 (6·9)	25·4 (7·3)
Gravidity				
1	25·4 (36)	19·6 (9)	25·0 (13)	31·8 (14)
2	17·6 (25)	17·4 (8)	11·5 (6)	25·0 (11)
≥3	57·0 (81)	63·0 (29)	63·5 (33)	43·2 (19)
Parity				
0	31·7 (45)	30·4 (14)	30·8 (16)	34·1 (15)
1	19·7 (28)	23·9 (11)	11·5 (6)	25·0 (11)
≥2	48·6 (69)	45·7 (21)	57·7 (30)	40·9 (18)
Smoking	25·4 (36)	30·4 (14)	23·1 (12)	22·7 (10)
Betel nut	53·5 (76)	54·3 (25)	55·8 (29)	50·0 (22)
Ethnicity				
Karen	83·8 (119)	84·8 (39)	80·8 (42)	86·4 (38)
Burmese	12·0 (17)	10·9 (5)	15·4 (8)	9·1 (4)
Others	4·2 (6)	4·3 (2)	3·8 (2)	4·5 (2)
Height (cm)	151·6 (5·3)	151·9 (4·7)	151·8 (5·9)	151·1 (5·0)
Weight (kg)	52·2 (8·0)	52·3 (9·5)	52·3 (6·9)	52·1 (7·7)
BMI (kg/m^2)	22·7 (3·2)	22·6 (3·8)	22·7 (2·5)	22·8 (3·2)
Fever (temperature ≥37·5)	46·5 (66)	43·5 (20)	46·2 (24)	50·0 (22)
Fever (including history of fever)	82·4 (117)	76·1 (35)	84·6 (44)	86·4 (38)
Duration of fever (day)	2 (0-7)	1 (0-7)	2 (0-7)	2 (0-7)
Haematocrit (%)	31·1 (4·6)	30·2 (4·8)	31·4 (4·7)	31·5 (4·4)
Anaemia				
no anaemia	68·3 (97)	58·7 (27)	71·2 (37)	75·0 (33)
moderate	31·7 (45)	41·3 (19)	28·8 (15)	25·0 (11)
severe	0·0 (0)	0·0 (0)	0·0 (0)	0·0 (0)
First recorded malaria in pregnancy	88·7 (126)	91·3 (42)	80·8 (42)	95·5 (42)
Pf parasitaemia (/uL)	8164 (16-207994)	7159 (16-207994)	10111 (16-124595)	8290 (96-120199)
Presence of Pf gametocytes	15·5 (22)	23·9 (11)	9·6 (5)	13·6 (6)

AL+: artemether-lumefantrine extended regimen, ASMQ: artesunate-mefloquine, BMI: body mass index, DP: dihydroartemisinin-piperaquine, EGA: estimated gestational age, Pf: *Plasmodium falciparum*.

S4 Table. The baseline characteristics of patients with *Plasmodium vivax* mono-infection

Baseline characteristic	Mean (SD), Percentage (n), Median (range)			
	All (n=353)	DP (n=121)	ASMQ (n=112)	AL+ (n=120)
EGA (week)	26·3 (8·1)	26·5 (7·9)	26·6 (8·4)	25·9 (8·0)
Trimester				
1st	8·5 (30)	6·6 (8)	8·0 (9)	10·8 (13)
2nd	44·2 (156)	45·5 (55)	45·5 (51)	41·7 (50)
3rd	47·3 (167)	47·9 (58)	46·4 (52)	47·5 (57)
Age (years)	25·6 (6·7)	25·6 (6·5)	25·9 (6·6)	25·2 (6·9)
Gravidity				
1	36·0 (127)	39·7 (48)	28·6 (32)	39·2 (47)
2	20·1 (71)	17·4 (21)	25·0 (28)	18·3 (22)
≥3	43·9 (155)	43·0 (52)	46·4 (52)	42·5 (51)
Parity				
0	42·5 (150)	44·6 (54)	35·7 (40)	46·7 (56)
1	19·0 (67)	19·8 (24)	22·3 (25)	15·0 (18)
≥2	38·5 (136)	35·5 (43)	42·0 (47)	38·3 (46)
Smoking	17·6 (62)	14·0 (17)	17·9 (20)	20·8 (25)
Betelnut	46·7 (165)	49·6 (60)	42·0 (47)	48·3 (58)
Ethnicity				
Karen	71·4 (252)	70·2 (85)	71·4 (80)	72·5 (87)
Burmese	22·1 (78)	20·7 (25)	24·1 (27)	21·7 (26)
Others	6·5 (23)	9·1 (11)	4·5 (5)	5·8 (7)
Height (cm)	150·9 (5·4)	150·9 (5·5)	150·9 (5·2)	150·9 (5·6)
Weight (kg)	51·5 (7·4)	52·3 (7·9)	51·8 (7·6)	50·5 (6·7)
BMI (kg/m2)	22·6 (3·0)	22·9 (3·0)	22·7 (3·1)	22·2 (2·7)
Fever (temperature ≥37·5)	21·0 (74)	20·7 (25)	20·5 (23)	21·7 (26)
Fever (including history of fever)	59·5 (210)	63·6 (77)	58·0 (65)	56·7 (68)
Duration of fever (day)	1 (0-10)	1 (0-10)	1 (0-7)	1 (0-10)
Haematocrit (%)	32·6 (3·7)	32·8 (3·6)	32·7 (3·7)	32·2 (3·7)
Anaemia				
no anaemia	83·9 (296)	87·6 (106)	83·9 (94)	80·0 (96)
moderate	16·1 (57)	12·4 (15)	16·1 (18)	20·0 (24)
severe	0·0 (0)	0·0 (0)	0·0 (0)	0·0 (0)
First recorded malaria in pregnancy	78· 5 (277)	73·6 (89)	83·9 (94)	78·3 (94)
Pv parasitaemia (/uL)	544 (16-83524)	576 (16-83524)	544 (16-29893)	512 (16-40694)
Presence of Pv gametocytes	63·7 (225)	66·1 (80)	56·3 (63)	68·3 (82)

AL+: artemether-lumefantrine extended regimen, ASMQ: artesunate-mefloquine, BMI: body mass index, DP: dihydroartemisinin-piperaquine, EGA: estimated gestational age, Pv: *Plasmodium vivax*.

S5 Table. The baseline characteristics of patients with *Plasmodium falciparum* infection, including both *P. falciparum* mono-infection and co-infection of *P. falciparum* and *Plasmodium vivax*

Baseline characteristic	Mean (SD), Percentage (n), Median (range)			
	All (n=157)	DP (n=50)	ASMQ (n=57)	AL+ (n=50)
EGA (week)	24·7 (8·2)	23·4 (7·9)	25·8 (8·7)	24·7 (7·9)
Trimester				
1st	10·2 (16)	10·0 (5)	10·5 (6)	10·0 (5)
2nd	51·0 (80)	58·0 (29)	40·4 (23)	56·0 (28)
3rd	38·9 (61)	32·0 (16)	49·1 (28)	34·0 (17)
Age (years)	26·1 (7·1)	25·9 (7·6)	27·1 (6·8)	25·0 (7·0)
Gravidity				
1	27·4 (43)	22·0 (11)	24·6 (14)	36·0 (18)
2	17·2 (27)	18·0 (9)	12·3 (7)	22·0 (11)
≥3	55·4 (87)	60·0 (30)	63·2 (36)	42·0 (21)
Parity				
0	33·8 (53)	32·0 (16)	29·8 (17)	40·0 (20)
1	19·1 (30)	24·0 (12)	12·3 (7)	22·0 (11)
≥2	47·1 (74)	44·0 (22)	57·9 (33)	38·0 (19)
Smoking	24·8 (39)	30·0 (15)	24·6 (14)	20·0 (10)
Betelnut	52·9 (83)	56·0 (28)	52·6 (30)	50·0 (25)
Ethnicity				
Karen	83·4 (131)	84·0 (42)	82·5 (47)	84·0 (42)
Burmese	12·1 (19)	10·0 (5)	14·0 (8)	12·0 (6)
Others	4·5 (7)	6·0 (3)	3·5 (2)	4·0 (2)
Height (cm)	151·4 (5·4)	152·1 (4·8)	151·5 (6·0)	150·5 (5·3)
Weight (kg)	52·2 (8·0)	52·8 (9·3)	52·4 (7·0)	51·4 (7·8)
BMI (kg/m2)	22·8 (3·1)	22·8 (3·7)	22·8 (2·6)	22·7 (3·0)
Fever (temperature ≥37·5)	45·9 (72)	40·0 (20)	43·9 (25)	54·0 (27)
Fever (including history of fever)	82·2 (129)	74·0 (37)	84·2 (48)	88·0 (44)
Duration of fever (day)	2 (0-7)	1 (0-7)	2 (0-7)	2 (0-7)
Haematocrit (%)	31·2 (4·5)	30·5 (4·7)	31·4 (4·6)	31·5 (4·3)
Anaemia				
no anaemia	69·4 (109)	62·0 (31)	71·9 (41)	74·0 (37)
moderate	30·6 (48)	38·0 (19)	28·1 (16)	26·0 (13)
severe	0·0 (0)	0·0 (0)	0·0 (0)	0·0 (0)
First recorded malaria in pregnancy	87·3 (137)	88·0 (44)	79·0 (45)	96·0 (48)
Species				
Pf mono-infection	90·4 (142)	92·0 (46)	91·2 (52)	88·0 (44)
Pf & Pv coinfection	9·6 (15)	8·0 (4)	8·8 (5)	12·0 (6)
Pf parasitaemia (/uL)	8541 (16-207994)	7159 (16-207994)	6782 (16-124595)	9608 (96-120199)
Pv parasitaemia (/uL)*	288 (16-12811)	104 (16-128)	1760 (32-6080)	1296 (16-12811)
Presence of Pf gametocytes	15·3 (24)	24·0 (12)	10·5 (6)	12·0 (6)
Presence of Pv gametocytes	5·1 (8)	2·0 (1)	5·3 (3)	8·0 (4)

AL+: artemether-lumefantrine extended regimen, ASMQ: artesunate-mefloquine, BMI: body mass index, DP: dihydroartemisinin-piperaquine, EGA: estimated gestational age, Pf: *Plasmodium falciparum*, Pv: *Plasmodium vivax*.

* Numbers are among those who had co-infection of Pv.

S6 Table. Cumulative proportion of fever and parasite clearance of falciparum and vivax for each treatment arm among pregnant women with history of fever or documented fever at enrolment

	Cumulative percentage of women without fever or asexual parasitaemia (95% CI) estimated by Kaplan-Meier method				
	All	AL+	ASMQ	DP	p-value
**Fever clearance**					
P. falciparum mono-infection	N=116	N=38	N=44	N=34	0·29
Day 1	74·1% (65·9-91·7)	71·1% (56·4-84·3)	81·8% (69·3-91·5)	67·7% (52·0-82·4)	
Day 2	99·1% (95·6-99·9)	97·4% (88·2-99·8)	100%	100%	
Day 3	100%	100%	100%	100%	
P. vivax mono-infection	N=210	N=68	N=65	N=77	0·29
Day 1	96·2% (93·0-98·2)	94·1% (86·8-98·1)	95·4% (88·3-98·8)	98·7% (93·8-99·9)	
Day 2	99·1% (96·9-99·8)	98·5% (93·0-99·9)	98·5% (92·7-99·9)	100%	
Day 3	100%	100%	100%	100%	
P. falciparum (all)*	N=128	N=44	N=49	N=38	0·09
Day 1	72·7% (64·8-80·1)	65·9% (52·0-79·3)	83·3% (71·6-92·2)	66·7% (51·4-81·2)	
Day 2	99·2% (96·0-99·9)	97·7% (89·7-99·8)	100%	100%	
Day 3	100%	100%	100%	100%	
P. vivax (all)*	N=222	N=74	N=70	N=81	0·11
Day 1	94·1% (90·5-96·7)	89·2% (80·9-95·0)	95·7% (89·0-98·9)	97·5% (92·1-99·5)	
Day 2	99·1% (97·0-99·8)	98·7% (93·5-99·9)	98·6% (93·1-99·9)	100%	
Day 3	100%	100%	100%	100%	
**Parasite clearance**					
*P. falciparum**	N=156	N=50	N=57	N=49	0·20
Day 1	15·4% (10·6-22·1)	8·0% (3·1-19·9)	17·5% (9·9-30·2)	20·4% (11·5-34·6)	
Day 2	44·5% (37·0-52·6)	42·0% (29·8-56·8)	42·1% (30·6-55·9)	49·7% (36·7-64·5)	
Day 3	71·9% (64·6-78·8)	65·6% (52·3-78·6)	75·4% (63·8-85·7)	73·8% (60·7-85·3)	
Day 4	89·3% (83·7-93·6)	82·8% (70·9-91·9)	89·5% (80·0-95·7)	95·6% (86·9-99·2)	
Day 5	98·0% (94·7-99·5)	97·9% (90·2-99·8)	98·3% (91·8-99·9)	97·8% (90·0-99·8)	
*P. vivax**	N=366	N=126	N=117	N=123	0·36
Day 1	64·8% (59·9-69·6)	62·7% (54·3-71·1)	65·8% (57·2-74·3)	65·9% (57·5-74·1)	
Day 2	95·4% (92·9-97·2)	92·1% (86·5-95·9)	96·6% (92·1-98·9)	97·6% (93·6-99·3)	
Day 3	99·7% (98·5-100)	99·2% (96·0-99·9)	100%	100%	

AL+: artemether-lumefantrine extended regimen, ASMQ: artesunate-mefloquine, DP: dihydroartemisinin-piperaquine.
*including co-infection of P. falciparum and P. vivax.

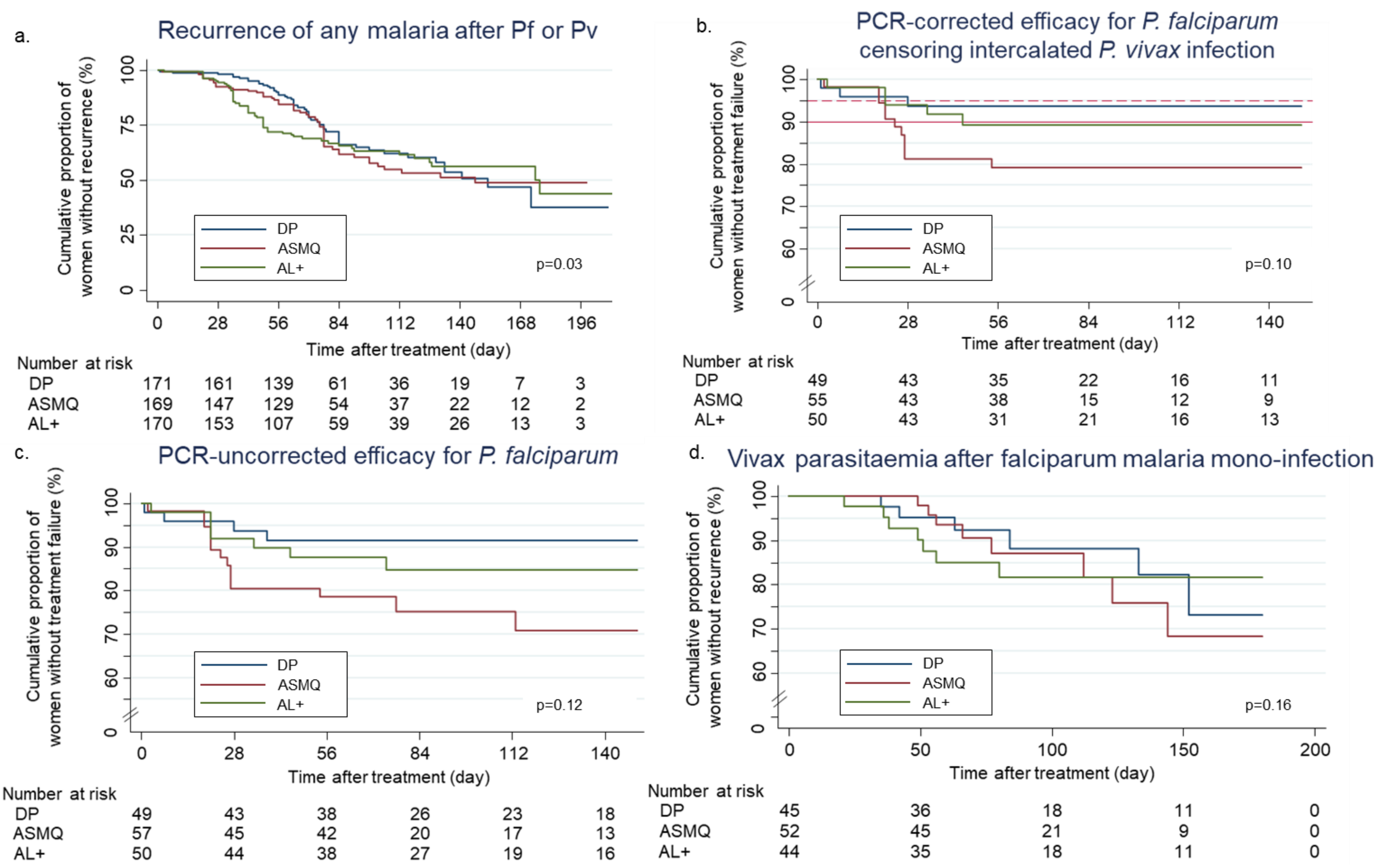

S2 Figure. Kaplan-Meier survival curves for antimalarials in pregnancy: Panel a. Recurrence of any malaria species. Panel b. uncomplicated *P. falciparum* in PCR-corrected efficacy censoring intercalated vivax malaria, Panel c. PCR-uncorrected efficacy of antimalarials, and Panel d. vivax malaria after falciparum mono-infection. p-values by Wilcoxon test.

a.

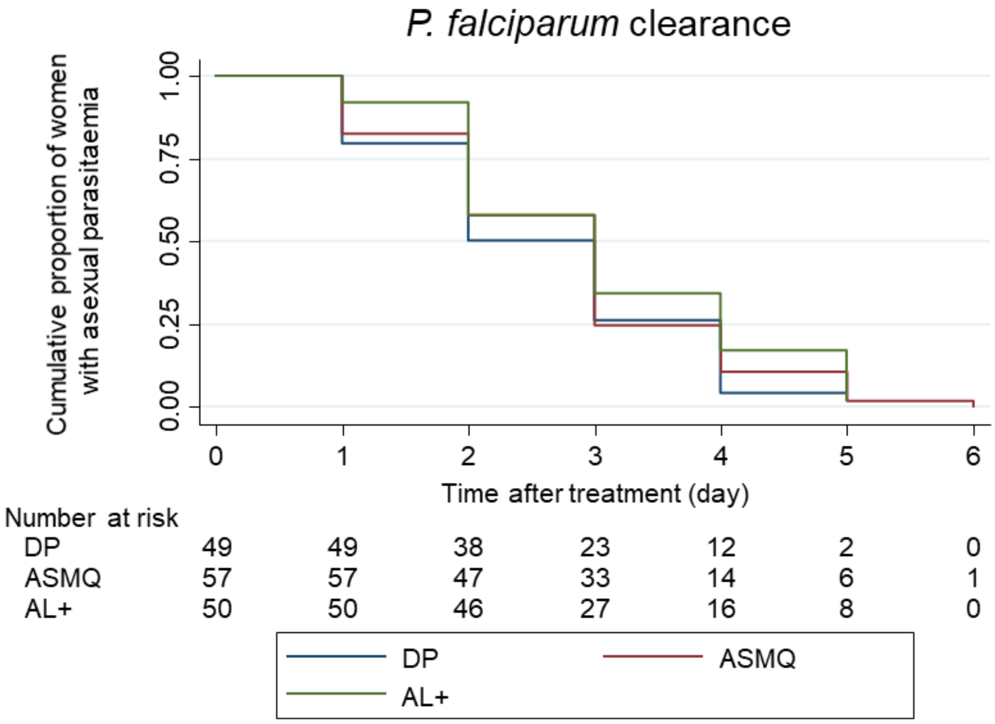

b.

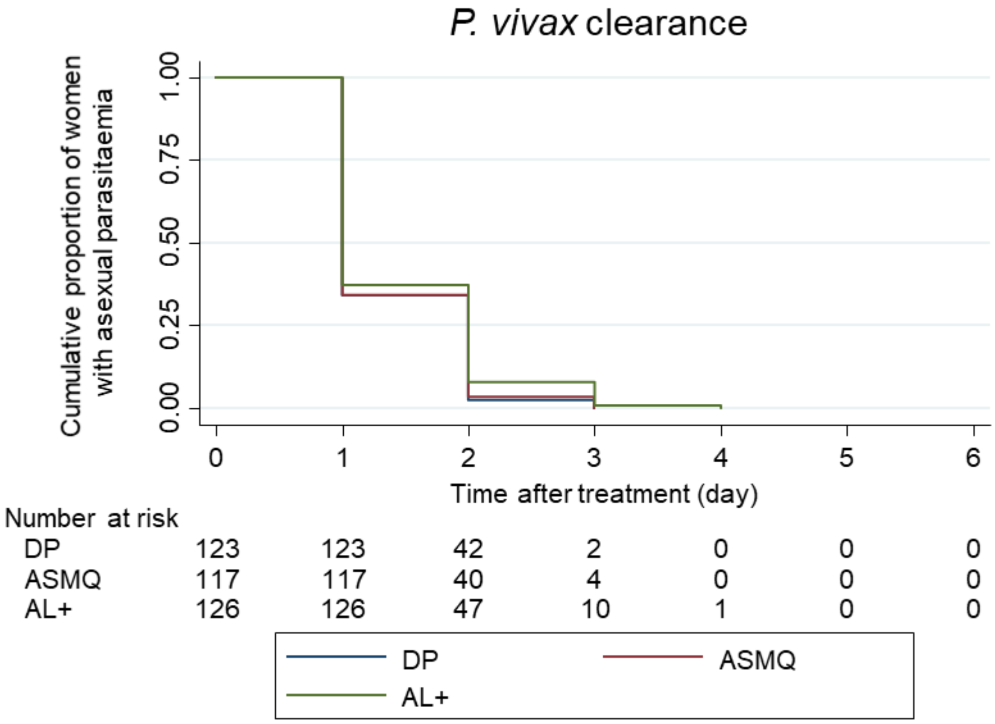

S3 Figure. Kaplan-Meier survival curves for parasite clearance of *Plasmodium falciparum* (Panel a) or *Plasmodium vivax* (Panel b) for each treatment arm.

S7 Table. Description of gametocyte carriage, congenital and placental malaria

**Gametocyte carriage**
There was no difference in the proportion of patients with gametocytaemia at presentation among the treatment arms either in falciparum or vivax malaria (**Table 1**). The gametocyte carriage in the 4 weeks after treatment of falciparum malaria was highest for ASMQ 78·3 (95% CI 35·8 to 148·6) per 1000 person-weeks followed by DP 19·2 (2·3 to 69·5) and AL^{+} 16·4 (2·0 to 59·2), based on very small proportions of patients with gametocytaemia during follow-up (DP 2/104 weeks, ASMQ 9/115 weeks, AL^{+} 2/122 weeks).
**Congenital and placental malaria**
Malaria smears of maternal peripheral blood, cord blood, placental blood, and/or the baby's peripheral blood were assessed in 76·7% (359/468) of women who had been followed until delivery. Of these 95·3% (342/359) were assessed for at least maternal peripheral blood and baby's peripheral blood, and 89·4% (321/359) for all four specimens. Rarely, the maternal peripheral smear was positive for *P. vivax* (4·0%, 14/353) or *P. falciparum* trophozoites (0·9%, 3/353). *P. vivax* trophozoites were found in the cord blood in one case (0·3%, 1/329) and the peripheral blood at delivery in another (0·3%, 1/348). In both cases, maternal peripheral blood was negative. Placental blood was positive for *P. falciparum* trophozoites in one case (0·3%, 1/333) and *P. vivax* trophozoites in three cases (0·9%, 3/333) and the maternal peripheral smear was positive for the same species in all of them. All three women with vivax placental malaria had three or four episodes of vivax in pregnancy. The woman with falciparum placental malaria had one vivax (27 days before delivery treated with ASMQ) and no falciparum malaria in her 85 days of follow-up during pregnancy.

S8 Table. Univariable and multivariable analyses of the risk of PCR-corrected treatment failure in pregnant women with uncomplicated falciparum malaria using a Cox proportional hazard model

Characteristic	Number of failure / assessed	Univariable*		Multivariable	
		HR (95% CI)	p-value	HR (95% CI)	p-value
Treatment					
DP	3/49	Reference		Reference	
ASMQ	11/55	3.27 (0.91-11.73)	0.07	3.00 (0.84-10.77)	0.09
AL+	6/50	1.91 (0.48-7.65)	0.36	1.85 (0.46-7.40)	0.39
Study year (year)	20/154	1.20 (0.82-1.76)	0.35		
EGA (week)	20/154	1.04 (0.98-1.10)	0.19		
Age (year)	20/154	1.07 (1.01-1.13)	0.03	1.06 (1.00-1.13)	0.04
Gravidity					
1	3/43	0.47 (0.13-1.67)	0.24		
2	4/26	1.20 (0.38-3.75)	0.75		
≥3	13/85	Reference			
Height (cm)	20/154	1.08 (0.99-1.17)	0.07		
Weight (kg)	20/154	1.03 (0.98-1.09)	0.28		
BMI (kg/m^2)	20/154	1.02 (0.89-1.18)	0.75		
Ethnic group					
Karen	16/130	Reference			
Burmese	4/17	1.89 (0.63-5.67)	0.26		
Others	0/7	No data	-		
Fever	10/69	1.25 (0.52-3.02)	0.62		
no fever	10/85	Reference			
Days of fever	20/154	1.04 (0.80-1.37)	0.75		
Haematocrit on day 0 (%)	20/154	1.02 (0.93-1.13)	0.63		
Anaemia					
no anaemia	16/106	Reference			
moderate	4/48	0.58 (0.19-1.75)	0.33		
Previous Pf episode	3/13	2.05 (0.58-7.25)	0.26		
No previous Pf	17/141	Reference			
Species					
Pf mono-infection	19/139	Reference			
Pf & Pv coinfection	1/15	0.45 (0.06-3.37)	0.44		
Presence of gametocytes	2/24	0.71 (0.16-3.07)	0.65		
No gametocytes	18/130	Reference			
Pf parasitaemia (log_{10}/µL)	20/154	1.71 (1.00-2.93)	0.05	1.71 (0.98-2.99)	0.06

AL+: extended regimen artemether-lumefantrine, ASMQ: Artesunate-mefloquine, BMI: body mass index, CI: confidence interval, DP: Dihydroartemisinin-piperaquine, EGA: estimated gestational age, HR: hazard ratio, Pf: *Plasmodium falciparum*, Pv: *Plasmodium vivax*. Global test for proportional-hazard assumption: p=0.34

* Adjusted for treatment groups.

S9 Table. Univariable and multivariable analyses of the risk of PCR-corrected treatment failure in pregnant women with uncomplicated falciparum malaria using a Cox proportional hazard model with *pfkelch13* mutations

Characteristic	Number of failure / assessed	Univariable*		Multivariable	
		HR (95% CI)	p-value	HR (95% CI)	p-value
Age (year)	20/154	1·07 (1·01-1·13)	0·03	1·06 (0·99-1·12)	0·08
Pf parasitaemia (log10/µL)	20/154	1·71 (1·00-2·93)	0·05	1·81 (1·00-3·28)	0·05
Treatment					
DP	3/49	Reference		Reference	
ASMQ	11/55	3·27 (0·91-11·73)	0·07	3·53 (0·97-12·87)	0·06
AL+	6/50	1·91 (0·48-7·65)	0·36	1·78 (0·45-7·14)	0·41
pfkelch13					
wild type	1/43	Reference		Reference	
any mutations	18/84	12·36 (1·64-93·17)	0·02	13·63 (1·80-102·95)	0·01

AL+: extended regimen artemether-lumefantrine, ASMQ: Artesunate-mefloquine, CI: confidence interval, DP: Dihydroartemisinin-piperaquine, HR: hazard ratio, Pf: Plasmodium falciparum.

* Adjusted for treatment groups.

a.

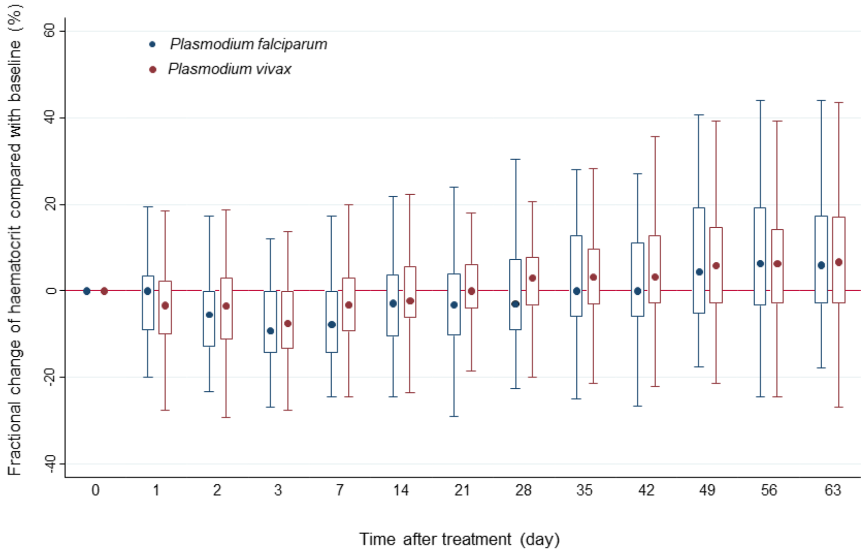

b.

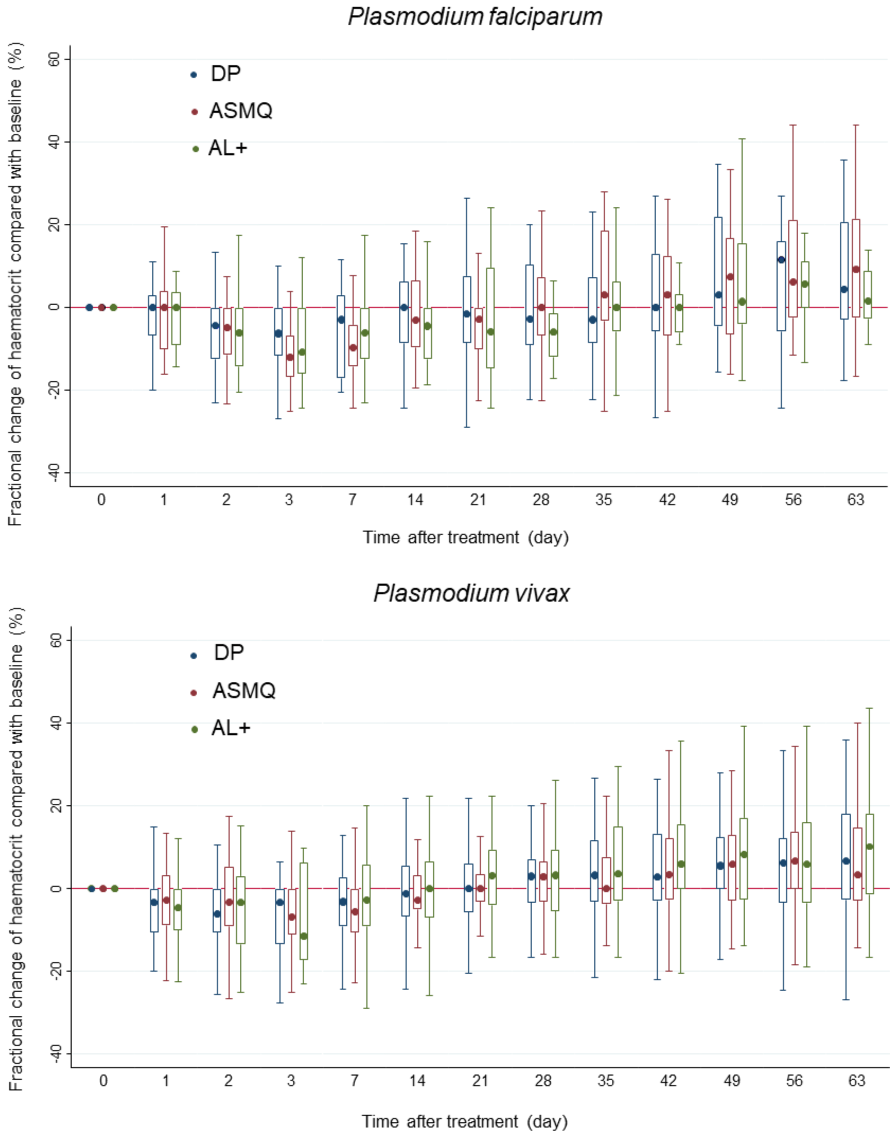

S4 Figure. Fractional change in haematocrit from baseline during the follow-up for each species (Panel a) or treatment stratified by malaria species (Panel b)

S10 Table. Prevalence of symptoms before and after treatment

Symptom	Proportion of women who had symptoms (n)			
	AL+	ASMQ	DP	p-value
Day 0				
Headache	78·6% (132/168)	79·9% (135/169)	78·4% (134/171)	
Muscle pain	55·4% (93/168)	50·0% (84/168)	55·0% (94/171)	
Joint pain	54·2% (91/168)	52·1% (88/169)	53·2% (91/171)	
Abdominal pain	19·1% (32/168)	22·5% (38/169)	20·5% (35/171)	
Anorexia	41·3% (69/167)	47·9% (81/169)	42·7% (73/171)	
Nausea	33·3% (56/168)	30·8% (52/169)	35·1% (60/171)	
Vomit	16·7% (28/168)	20·7% (35/169)	17·5% (30/171)	
Itchiness	8·9% (15/168)	11·8% (20/169)	9·9% (17/171)	
Rash	4·8% (8/168)	4·7% (8/169)	4·7% (8/171)	
Urticaria	0·0% (0/168)	0·6% (1/169)	0·0% (0/171)	
Dizziness	66·1% (111/168)	58·6% (99/169)	62·0% (106/171)	
Tinnitus	25·6% (43/168)	22·5% (38/169)	25·2% (43/171)	
Hearing abnormality	4·8% (8/168)	1·8% (3/169)	7·0% (12/171)	
Abnormal vision	8·9% (15/168)	7·1% (12/169)	10·5% (18/171)	
Diarrhoea	3·0% (5/168)	0·6% (1/169)	2·3% (4/171)	
Palpitation	34·5% (58/168)	26·6% (45/169)	35·1% (60/171)	
Fatigue	45·8% (77/168)	50·9% (86/169)	47·4% (81/171)	
Sleep disturbance	40·5% (68/168)	36·7% (62/169)	29·8% (51/171)	
Confusion	0·0% (0/168)	0·0% (0/169)	0·6% (1/171)	
Numbness	19·1% (32/168)	13·6% (23/169)	23·4% (40/171)	
Contraction	1·2% (2/168)	1·2% (2/166)	1·2% (2/169)	
Absence of fetal movement	0·0% (0/149)	1·3% (2/152)	2·0% (3/154)	
Vaginal bleeding	0·6% (1/168)	0·0% (0/169)	0·0% (0/171)	
Day 1-14*				
Headache	13·9% (5/36)	17·7% (6/34)	16·2% (6/37)	0·95
Muscle pain	2·7% (2/75)	2·4% (2/84)	6·5% (5/77)	0·44
Joint pain	3·9% (3/77)	3·7% (3/81)	3·8% (3/80)	1·00
Abdominal pain	5·9% (8/136)	2·3% (3/131)	2·2% (3/135)	0·20
Anorexia	2·0% (2/98)	5·7% (5/88)	3·1% (3/97)	0·40
Nausea	4·5% (5/112)	13·7% (16/117)	2·7% (3/110)	0·004
Vomit	0·7% (1/140)	13·4% (18/134)	1·4% (2/140)	<0·0001
Itchiness	2·6% (4/153)	0·7% (1/149)	1·3% (2/153)	0·52
Rash	1·9% (3/160)	0·0% (0/161)	0·6% (1/162)	0·13
Urticaria	0·6% (1/168)	0·6% (1/168)	0·0% (0/170)	0·55
Dizziness	5·3% (3/57)	37·1% (26/70)	10·8% (7/65)	<0·0001
Tinnitus	1·6% (2/125)	3·8% (5/131)	3·1% (4/128)	0·64
Hearing abnormality	0·0% (0/160)	0·0% (0/166)	0·0% (0/158)	-
Abnormal vision	2·0% (3/153)	0·6% (1/157)	0·0% (0/152)	0·23
Diarrhoea	2·5% (4/163)	1·2% (2/168)	1·8% (3/166)	0·59
Palpitation	0·9% (1/110)	2·4% (3/124)	0·9% (1/111)	0·63
Fatigue	5·5% (5/91)	2·4% (2/83)	4·5% (4/89)	0·64
Sleep disturbance	5·0% (5/100)	14·0% (15/107)	5·8% (7/120)	0·04
Confusion	0·0% (0/168)	0·0% (0/169)	0·6% (1/169)	1·00
Numbness	0·7% (1/136)	1·4% (2/146)	1·5% (2/130)	0·87
Contraction	1·8% (3/165)	1·2% (2/163)	0·6% (1/165)	0·71
Absence of fetal movement	0·7% (1/147)	0·0% (0/148)	0·7% (1/149)	0·78
Vaginal bleeding	0·6% (1/166)	2·4% (4/168)	1·8% (3/169)	0·50

AL+: artemether-lumefantrine extended regimen, ASMQ: artesunate-mefloquine, DP: dihydroartemisinin-piperaquine. p-values by Fisher's exact test.

* Women who had the symptom on day 0 are excluded.

S11 Table. Description of pregnancy outcomes

Twins	There were three twin pregnancies (one in DP and two in AL^+) and among the twins, one of the six newborns were stillborn (in AL+). All twin births are excluded from description of other birth outcomes.
Miscarriage	There were 280 pregnant women enrolled before 28 weeks of gestation of whom 266 were followed up at least until 28 weeks of gestation: and three miscarriages (1·1%, 3/266), one in each treatment group (1/91 in DP, 1/85 in ASMQ, 1/90 in AL^+) (**S9 Table**).
Stillbirth	Of 462 singleton births (excluding twins, miscarriage and lost to follow-up), five were stillborn (1·1%, 5/462): one in DP (1/155), two each in ASMQ (2/151) and AL^+ (2/156) (**S9Table**).
Congenital abnormality	Five newborns had congenital abnormalities (1·1%, 5/462), three AL^+ and two DP. None of these had been exposed to first trimester malaria treatment **(S10Table).**
Neonatal death	There were nine neonatal deaths: three AL^+, four DP and two ASMQ. Six were related to prematurity (including one due to placental abruption), one resulted from intrapartum asphyxia due to tight nuchal umbilical cord with severe intrauterine growth restriction (2·5th centile), one had a congenital diaphragmatic hernia, and one had meconium aspiration syndrome (10th centile) **(S111Table).**
Preterm birth (PTB)	The overall proportion of preterm birth was 8·3% (35/423) and was not different among different treatment groups (p=0·45). Treatment was not associated with estimated gestational age at birth by univariable and multivariable linear regression (**S12 Table**).
Small for gestational age (SGA)	Birthweight of 397 (88%) normal live singletons was measured within 72 hours; 26·2% (104) were small for gestational age and proportions were not different among the treatment groups.<br><br>Length, arm circumference, head circumference at birth and Apgar score at 5 min was also not different among three treatment groups.

S12 Table. Details of pregnant women who had miscarriage or stillbirth

Outcome	Clinical details of events surrounding birth outcome
**DP group**	
Miscarriage	18y, G1P0, symptomatic vivax, EGA 12+0, 3 days fever, FHB present on day 3 and discharged. At 13+3 febrile illness, admitted for fever 2 days and treated conservatively – suspected viral illness. EGA 22+2, d77 post DP asymptomatic vivax, treated with chloroquine, day 2 start suprapubic pain and back pain and miscarriage day 3 at EGA 22+4, BW 520g, 300g placenta; FHB lost just before products expelled.
Stillbirth –intrapartum	28y, G6P5, symptomatic falciparum, EGA 20+3, 2 days fever, BT 38·9°C; At EGA 25+3 and 31+3 recurrent asymptomatic vivax. At EGA 35+0 arrived in shock (BP 60/20 mmHg, FHR 108), severe watery diarrhoea (suspected cholera), resuscitated, discharged after 7 days. EGA 35+6 repeat asymptomatic vivax. Three days before labour, she had normal ANC visit. Arrived 38+0, no FHB present, antepartum haemorrhage, placental abruption, post-partum haemorrhage. Normal male, BW 2690g, 16th centile weight for gestation, no maceration, syphilis negative.
**ASMQ group**	
Miscarriage	18y, G2P0, symptomatic falciparum 2·8% iRBC, EGA 7+1, 5 days fever, BT>39°C, plus pyelonephritis, plus stool test revealed hookworm, *Ascaris lumbricoides* and *Trichuris trichuria* [treatment delayed until out of first trimester].FHB present on day of discharge D7. Day 53 vivax malaria 0·3% iRBC EGA 14+5 no FHB detected, treated with chloroquine, plus urine culture for *Escherichia coli*. The miscarriage complete EGA 15+0.
Stillbirth –prepartum<br><br>*And maternal mortality*	26y, G3P2, symptomatic vivax (0·5% parasitaemia) EGA 36+6, 7 days fever T 38·4°C; rapidly responded to treatment, clearing her blood stage parasites within 48 hours. The appeared well, ambulatory, FHB normal and due for discharge but became unwell with a rapidly progressive acute respiratory distress syndrome (ARDS) requiring ventilation three days (67 hours) into treatment. Labour was induced in ICU for absent FHB and delivered by forceps, normal BW 2500 g male 49th centile. Ventilatory requirements increased and the patient died on day 7. Concomitant infections (TB, HIV, syphilis) were excluded.
Stillbirth –intrapartum	19y, G2P0, asymptomatic vivax EGA 11+5, normal ANC care, last seen EGA 38+0 weeks with normal obstetric and physical exam. At EGA 38+3 homebirth,

	with a traditional birth attendant, long labour, lives far and difficult to reach SMRU. Normal male weight not available, no maceration.
**AL+ group**	
Miscarriage	40y, G9P8, symptomatic vivax, EGA 15+0, 2 days fever, treatment finished d4, at midnight on day 5, EGA miscarriage 15+4 at 01:30 am [fetus is anencephalic and with an abdominal wall defect]. The patient had a 10% drop in haematocrit but not transfused, oral haematinics only.
Stillbirth –prepartum	33y, G7P5 (4 alive, 1 died 9-month-age fever, 1 miscarriage), symptomatic falciparum (0·1% iRBC), EGA 37+0, 7 days fever BT 38·1°C; rapid recover with treatment. Day 21 no FHB detected at routine ANC care and spontaneous delivery occurred the next day at EGA 40+0· Mother thought baby was moving but at delivery the fetus was macerated indicating fetal death in utero at least 2-3 days previously. Normal BW 2800g female 10th centile for gestation, syphilis negative.
Stillbirth –intrapartum	38y, G6P5, asymptomatic vivax EGA 17+4, smoker since age 7; EGA 31+2 normal obstetric and physical exam, 3 days later presented APH, managed conservatively, next day PPROM, fetal distress, referred in emergency, stillbirth due to placental abruption at EGA 32+0· Normal BW 1600g female 41st centile

AL+ extended regimen artemether-lumefantrine; ANC antenatal care; ASMQ artesunate-mefloquine; BT body temperature; BW birthweight; DP dihydroartemisinin-piperaquine; EGA estimated gestational age; FHB fetal heart beat; FHR fetal heart rate; G gravidity; iRBC infected red blood cells; P parity; PPROM Preterm premature rupture of the membrane; SMRU Shoklo Malaria Research Unit; TB tuberculosis.

S13 Table. Details of congenital abnormality and ICD-10 coding

EGA at exposure	ICD-10 code	Description
**DP group**		
EGA 16+1 weeks	Q37.9	33y, G6P4, EGA 34+2, female, BW 1480g, 2nd centile, cleft lip and palate on right side
EGA 31+4 weeks	Q79.0	22y, G1P0, EGA 40+2, female, BW 2860g, 12th centile, diaphragmatic hernia confirmed by antenatal ultrasound, was admitted to special care baby unit after birth with a diagnosis of early onset neonatal sepsis and treated but deteriorated rapidly, and died 2 days of life
**AL+ group**		
EGA 14+4 weeks	Q37.9	20y, G1P0, EGA 40+0, female, BW 2895g, 16th centile, cleft lip and palate on left side
EGA 20+2 weeks	Q24.9	24y, G4P2, EGA 38+4, female, born at home, acyanotic congenital heart disease diagnosed clinically.
EGA 31+6 weeks	Q01.9 and Q70.0	40y, G9P8, EGA 33+5, male, BW 2030g, 38th centile, nasofrontal encephalocoele and multiple syndactyly

AL+ extended regimen artemether-lumefantrine; BW birthweight; DP dihydroartemisinin-piperaquine; EGA estimated gestational age; G gravidity; ICD-10 International statistical classification of diseases and related health problems, 10th version; P parity.

S14 Table. Details of neonatal mortality

Cause of death	Description
**DP group**	
Respiratory distress, prematurity	18y G1P0, PV symptomatic EGA 16+2; reappearance PV symptomatic day 76 treated with CHQ. Day 79 preterm labour at home, NVD EGA 28+1, normal live born female infant weighing 1120g at 02:30am. At daylight they presented and were admitted to the clinic. Baby passed away at 39 hours of active care.
Respiratory distress, congenital abnormality	22y, G1P0, symptomatic PV EGA 31+4, and spontaneous labour at term EGA 40+2, female, BW 2860g, 12th centile, diaphragmatic hernia confirmed by ultrasound, was admitted to special care baby unit after birth with a diagnosis of early onset neonatal sepsis and treated but deteriorated rapidly, and died at 2 days of life.
Intrapartum asphyxia with severe IUGR	19y,G3P1, symptomatic PV, EGA 35+3, with spontaneous labour at EGA 40+0, NVD, cord around the neck and arm, cord cut on perineum, failed resuscitation stopped at 20 minutes, no spontaneous respiration, male BW 2600g, APGAR score 2^1 and 4^5, 2nd·5th centile weight for age.
Meconium aspiration syndrome	19y G1P0, PV asymptomatic EGA 22+1, reappearance PV asymptomatic on day 84 and spontaneous labour on day 97 at EGA 40+2, with thick meconium, IUGR 10th centile, BW 2940, APGAR score 4^1 and 7^5 and cardio respiratory arrest 6 hours after birth; and could not be resuscitated.
**ASMQ group**	
Apnoea of prematurity (placental abruption)	20y, G3P2, PF symptomatic 2·2% iRBC, EGA 24+2, negative on d6; spontaneous labour on d11, arrived fully dilated, NVD live born 25+1 week normal male infant BW 730 g, APGAR score 1^1 and 2^5, who passed away after 70 mins of palliative care. Retained placenta delivered after 30 mins with 50 cc old retroplacental clot.
Apnoea of prematurity	19y, G3P1, labour pain, 2·4% iRBC d46, with spontaneous labour at home, no time to come, EGA 26+3, NVD, live born (weak cry), externally normal, died in <15 minutes.
**AL+ group**	
Apnoea of prematurity	42y, G7P4+2, PV asymptomatic EGA 15+2, reappearance PV symptomatic day 49, treated with CHQ. Day 11 of episode 2 she went into labour at EGA 24+5 (she had mild pneumonia treated with oral amoxycillin and salbutamol inhaler), and NVD, normal live born male BW 600g, APGAR score of 1^1 and 1^5, who passed away, 38 mins of palliative care.
Respiratory distress, prematurity	29y G2P1, asymptomatic PV EGA 28+5, discharged well at day 3 but presented 7 days later with pre-term labour with fetal distress; NVD, normal female, EGA

	29+6 weeks, BW 1300g, and APGAR score was 1^1 and 2^5, neonatal resuscitation was done but baby passed away after 40 minutes of life.
Prematurity	20y G2P1, symptomatic PV EGA 31+4, preterm labour commenced during treatment, dexamethasone (full course) and nifedipine commenced. She became afebrile but NVD, on day 3 while still on malaria treatment, EGA 32+0 weeks, BW 1720g female, APGAR score was 9^1 and 10^5, and admitted to the Special Care Baby Unit, gaining weight and starting to tolerate milk feeds but the mother discharged herself and the baby on day 7. The mother could not be encouraged to return to the clinic. The baby passed away at home on day 12 of life.

Abbreviations: BW birthweight; CHQ chloroquine; EGA estimated gestational age; G gravidity; iRBC infected red blood cells; IUGR intra-uterine growth restriction; NVD normal vaginal delivery; P parity; PF *Plasmodium falciparum*; PV *Plasmodium vivax*.

S15 Table. Univariable and multivariable linear regression analyses of the characteristics associated with gestational week at birth among those who had malaria in pregnancy

Characteristic	N	Univariable*		Multivariable	
		Coefficient (95% CI)	p-value	Coefficient (95% CI)	p-value
EGA at malaria episode (week)	452	0·014 (-0·01-0·04)	0·21		
Age (years)	452	0·02 (-0·01-0·05)	0·15		
Gravidity					
1	156	-0·12 (-0·53-0·30)	0·58		
2	86	-0·23 (-0·72-0·27)	0·37		
≥3	210	Reference			
Previous history of stillbirth	8	-1·36 (-2·75-0·03)	0·06	-1·38 (-2·76-0·002)	0·05
no	444	Reference		Reference	
Height (cm)	452	0·04 (0·01-0·07)	0·02		
Weight (kg)	452	0·03 (0·01-0·05)	0·01	0·03 (0·01-0·05)	0·01
BMI (kg/m^2)	452	0·05 (-0·01-0·11)	0·10		
MUAC (cm)	450	0·05 (-0·02-0·12)	0·15		
Ethnic group					
Karen	342	Reference			
Burmese	82	0·38 (-0·10-0·86)	0·12		
Others	28	0·33 (-0·43-1·10)	0·39		
Smoking	90	-0·28 (-0·74-0·17)	0·22		
non smoker	362	Reference			
Betel nuts use	224	0·03 (-0·34-0·40)	0·87		
no	228	Reference			
Fever	126	-0·12 (-0·53-0·29)	0·57		
no fever	326	Reference			
Days of fever	452	0·01 (-0·10-0·12)	0·87		
Haematocrit on day 0 (%)	452	0·016 (-0·03-0·06)	0·49		
Anaemia					
no anaemia	365	Reference			
moderate	87	-0·21 (-0·68-0·25)	0·36		
Species					
Pf mono-infection	128	Reference			
Pv mono-infection	309	0·04 (-0·37-0·45)	0·87		
Pf & Pv coinfection	14	0·30 (-0·79-1·40)	0·59		
Pm mono-infection	1	-0·69 (-4·61-3·23)	0·73		
Parasitaemia load					
Lowest	107	Reference			
Lower middle	112	0·06 (-0·46-0·59)	0·81		
Higher middle	114	0·10 (-0·42-0·63)	0·69		
Highest	119	-0·18 (-0·69-0·34)	0·51		
Treatment					
DP	152	Reference			
ASMQ	149	0·21 (-0·23-0·66)	0·35	0·26 (-0·18-0·71)	0·25
AL+	151	0·01 (-0·44-0·46)	0·96	0·08 (-0·36-0·52)	0·72
Total number of MiP	452	0·08 (-0·08-0·24)	0·34		

AL+: extended regimen artemether-lumefantrine, ASMQ: Artesunate-mefloquine, BMI: body mass index, CI: confidence interval, DP: Dihydroartemisinin-piperaquine, EGA: estimated gestational age, MUAC: middle upper arm circumference, Pf: *Plasmodium falciparum*, Pm: *Plasmodium malariae*, Pv: *Plasmodium vivax*.* Adjusted for treatment groups.

S16 Table. Univariable and multivariable linear regression analyses of the characteristics associated with SGA z-score (birthweight for gestational age and newborn sex at birth)

Characteristic	N	Univariable*		Multivariable	
		Coefficient (95% CI)	p-value	Coefficient (95% CI)	p-value
EGA at malaria episode (week)	397	0·002 (-0·01-0·01)	0·79		
Age (years)	397	0·01 (0·00-0·02)	0·15		
Gravidity					
1	140	-0·42 (-0·62--0·23)	<0·0001	-0·35 (-0·54- -0·17)	0·0002
2	78	0·02 (-0·21-0·26)	0·83	0·06 (-0·16-0·28)	0·60
≥3	179	Reference		Reference	
Previous history of stillbirth	8	-0·10 (-0·74-0·53)	0·74		
no	389	Reference			
Height (cm)	397	0·03 (0·01-0·05)	0·0003	0·03 (0·02-0·05)	<0·0001
Weight (kg)	397	0·04 (0·02-0·05)	<0·0001		
BMI (kg/m^2)	397	0·07 (0·04-0·10)	<0·0001	0·05 (0·02-0·08)	0·0005
MUAC (cm)	396	0·06 (0·02-0·09)	0·001		
Ethnic group					
Karen	299	Reference			
Burmese	73	-0·07 (-0·30-0·16)	0·56		
Others	25	0·13 (-0·24-0·50)	0·49		
Smoking	78	-0·16 (-0·38-0·06)	0·16		
non smoker	319	Reference			
Betel nuts use	197	0·12 (-0·06-0·30)	0·18		
no	200	Reference			
Fever	109	0·10 (-0·09-0·30)	0·30		
no fever	288	Reference			
Days of fever	397	-0·05 (-0·10-0·01)	0·10		
Haematocrit on day 0 (%)	397	0·02 (-0·01-0·04)	0·15		
Anaemia					
no anaemia	320	Reference		Reference	
moderate	77	-0·32 (-0·54--0·10)	0·01	-0·24 (-0·45- -0·03)	0·03
Species					
Pf mono-infection	102	Reference			
Pv mono-infection	280	-0·08 (-0·29-0·12)	0·43		
Pf & Pv coinfection	14	-0·06 (-0·56-0·44)	0·81		
Pm mono-infection	1	-0·07 (-1·84-1·71)	0·94		
Parasitaemia load					
Lowest	100	Reference			
Lower middle	87	-0·12 (-0·38-0·14)	0·37		
Higher middle	103	0·05 (-0·19-0·30)	0·67		
Highest	107	-0·09 (-0·33-0·16)	0·47		
Treatment					
DP	133	Reference		Reference	
ASMQ	129	-0·015 (-0·23-0·20)	0·89	-0·04 (-0·24-0·16)	0·72
AL+	135	0·01 (-0·21-0·22)	0·96	0·06 (-0·14-0·25)	0·56
Total number of MiP	397	-0·17 (-0·25--0·10)	<0·0001	-0·14 (-0·22- -0·07)	0·0001

AL+: extended regimen artemether-lumefantrine, ASMQ: Artesunate-mefloquine, BMI: body mass index, CI: confidence interval, DP: Dihydroartemisinin-piperaquine, EGA: estimated gestational age, MUAC: middle upper arm circumference, Pf: *Plasmodium falciparum*, Pm: *Plasmodium malariae*, Pv: *Plasmodium vivax*.* Adjusted for treatment groups.

S17 Table. Birth outcomes in pregnant women who had malaria and were enrolled in the first trimester

Baseline characteristic	Mean (SD), Percentage (n), Median (range)		
	DP	ASMQ	AL+
Enrolled	13	15	18
Lost	1	1	3
Miscarriage	1/12	1/14	0/16
Stillbirth	0/11	1/13	0/15
Congenital abnormality	0/11	0/13	0/15
EGA (week)	39·1 (34·1-40·4)	39·1 (34·3-40·4)	39·5 (33·4-40·6)
Preterm birth	1/11 (9%)	1/12 (8%)	3/15 (20%)
Birth weight weighted in 3 days	8/11	9/12	13/15
Birth weight (g)	2730 (1739-3390)	3170 (2500-3510)	2920 (1580-3750)
Small-for-gestational age	2/7 (29%)	1/9 (11%)	4/12 (33%)
Length (cm)	48 (45-53) (n=8)	50 (48-52) (n=9)	49 (43-56) (n=12)
Placental weight (g)	400 (190-580) (n=7)	568 (460-680) (n=8)	510 (250-912) (n=12)

AL+: artemether-lumefantrine extended regimen, ASMQ: artesunate-mefloquine, DP: dihydroartemisinin-piperaquine, EGA: estimated gestational age.
